# Impact of the COVID-19 Pandemic on the Global Delivery of Mental Health Services and Telemental Health: Systematic Review

**DOI:** 10.2196/38600

**Published:** 2022-08-22

**Authors:** Caroline Zangani, Edoardo G Ostinelli, Katharine A Smith, James S W Hong, Orla Macdonald, Gurpreet Reen, Katherine Reid, Charles Vincent, Rebecca Syed Sheriff, Paul J Harrison, Keith Hawton, Alexandra Pitman, Rob Bale, Seena Fazel, John R Geddes, Andrea Cipriani

**Affiliations:** 1 Department of Psychiatry University of Oxford Oxford United Kingdom; 2 Oxford Health NHS Foundation Trust Warneford Hospital Oxford United Kingdom; 3 Department of Experimental Psychology University of Oxford Oxford United Kingdom; 4 Division of Psychiatry University College London London United Kingdom

**Keywords:** COVID-19, coronavirus, mental health services, telemental health, telepsychiatry, face-to-face

## Abstract

**Background:**

The COVID-19 pandemic required mental health services around the world to adapt quickly to the new restrictions and regulations put in place to reduce the risk of transmission. As face-to-face contact became difficult, virtual methods were implemented to continue to safely provide mental health care. However, it is unclear to what extent service provision transitioned to telemental health worldwide.

**Objective:**

We aimed to systematically review the global research literature on how mental health service provision adapted during the first year of the pandemic.

**Methods:**

We searched systematically for quantitative papers focusing on the impact of the COVID-19 pandemic on mental health services published until April 13, 2021, in the PubMed, Embase, medRxiv, and bioXriv electronic bibliographic databases, using the COVID-19 Open Access Project online platform. The screening process and data extraction were independently completed by at least two authors, and any disagreement was resolved by discussion with a senior member of the team. The findings were summarized narratively in the context of each country’s COVID-19 Stringency Index, which reflects the stringency of a government’s response to COVID-19 restrictions at a specific time.

**Results:**

Of the identified 24,339 records, 101 papers were included after the screening process. Reports on general services (n=72) showed that several countries’ face-to-face services reduced their activities at the start of the pandemic, with reductions in the total number of delivered visits and with some services forced to close. In contrast, telemental health use rapidly increased in many countries across the world at the beginning of the pandemic (n=55), with almost complete virtualization of general and specialistic care services by the end of the first year. Considering the reported COVID-19 Stringency Index values, the increased use of virtual means seems to correspond to periods when the Stringency Index values were at their highest in several countries. However, due to specific care requirements, telemental health could not be used in certain subgroups of patients, such as those on clozapine or depot treatments and those who continued to need face-to-face visits.

**Conclusions:**

During the pandemic, mental health services had to adapt quickly in the short term, implementing or increasing the use of telemental health services across the globe. Limited access to digital means, poor digital skills, and patients’ preferences and individual needs may have contributed to differences in implementing and accessing telemental health services during the pandemic. In the long term, a blended approach, combining in-person and virtual modalities, that takes into consideration the needs, preferences, and digital skills of patients may better support the future development of mental health services. It will be required to improve confidence with digital device use, training, and experience in all modalities for both clinicians and service users.

## Introduction

At the start of the COVID-19 pandemic, mental health services around the world, along with health services in general, needed to adapt quickly to reduce the risk of infection and transmission while continuing to support those with mental health problems [1]. However, as identified early in the COVID-19 pandemic by the World Health Organization (WHO), the pandemic went beyond the physical threat to also affect mental health [2].

The link between COVID-19 and mental illness is increasingly supported by robust evidence. The association is bidirectional; mental illness increases the risk of subsequent COVID-19 infection, and the risk of a new mental health diagnosis increases up to 180 days following COVID-19 infection [3,4]. COVID-19 and related coronavirus infections, such as SARS and Middle East respiratory syndrome (MERS), are associated with increased subsequent risk of depression, anxiety, and other neuropsychiatric consequences, such as dysexecutive syndrome [5-7]. This is in addition to any indirect effects on mental health caused by restrictions and lockdowns imposed by the COVID-19 pandemic [8]. Unemployment, financial insecurity, and other socioeconomic effects of the COVID-19 pandemic also adversely impact mental health. Mental health consequences can occur both at the time of a crisis and afterwards [9]. For instance, it has been suggested that suicide rates in some countries may have a delayed increase as a consequence of the social, health, and economic disruption associated with the pandemic [9]. As the pandemic evolved, access to vaccines and their uptake have altered the pattern of infection [10], and therefore, restrictive measures adopted by public health institutions have changed [11]. Taken together, all these factors suggest that there will be a rise in mental health treatment needs, and this is likely to be long-lasting. Consequently, mental health services have also started to plan or implement measures to prepare for an anticipated increase in demand related both directly to COVID-19 infection and indirectly to the sequelae of its associated restrictions.

During the pandemic, the WHO proposed that the goals of mental health care services need to focus not only on responding to the acute mental health emergency, but also on recovery thereafter and preparations for future emergencies [2]. How and to what extent this has happened is yet to be seen.

In contrast to the volume of data on the mental health impact of COVID-19, there have been few systematic considerations of the response of mental health services. Some reports reflect consultation with experts and service users to consider what changes in services might be needed to meet the mental health consequences of COVID-19 [12]. Organizations, such as the WHO, have suggested an integrated approach including mental health and psychosocial support in the COVID-19 response [2], while the United Nations has highlighted the need for changes and investment immediately to reduce mental health effects later [13].

Preliminary data from a WHO survey suggested a considerable detrimental impact on mental health services [14], with telemental health (TMH) considered critical to maintaining delivery of mental health services in response to pandemic-related social distancing measures and confinements [15]. Prompted by this, several countries modified national telemedicine regulations to promote its spread [16]. However, it is unclear whether the apparently rapid transition to TMH observed in many high-income countries [1] has occurred globally, which services have been affected, and whether this change has been sustained through the different phases of the pandemic.

To understand the degree to which service provision has changed during the first year of the pandemic and the extend of the transition to TMH in different countries, we systematically reviewed the available literature on mental health services during the pandemic. We also assessed these changes across the different phases of the COVID-19 pandemic and in the context of the local restrictions imposed.

## Methods

### Overview

We performed a systematic review of studies describing the impact of the COVID-19 pandemic on mental health services up to 1 year after the pandemic declaration on March 11, 2020 [17]. This is part of a wider assessment of the impact of the COVID-19 pandemic across the mental health field (previously published protocol for the full project is available on PROSPERO, CRD42020178819) [18].

### Search Strategy and Screening Process

The search strategy for the full project (see above) included terms relating to mental health and the COVID-19 pandemic, SARS, and MERS, with specific and generic specifiers (eg, Medical Subject Headings [MeSH] terms; for full details, see Multimedia Appendix 1). We searched PubMed, Embase, medRxiv, and bioXriv electronic bibliographic databases using the COAP (COVID-19 Open Access Project) online platform [19] for relevant reports from inception until April 13, 2021. Records on SARS and MERS were independently searched on PubMed and Embase from inception to April 13, 2021.

At least two members of the review team (CZ, EGO, GR, JSWH, KR, KAS, OM, or RS) independently screened the title and abstract of the retrieved records. Full texts of the potentially eligible records were then independently assessed against the eligibility criteria. Any disagreement about eligibility was discussed with a third member of the research team (AC, AP, CV, or KH). The included articles and relevant systematic reviews were also screened for references to identify additional records. References from all sources were cross-checked to ensure that all referenced documents had been searched.

### Eligibility Criteria

To examine the consequences of the COVID-19 pandemic on mental health services, we included only reports providing quantitative data on changes in service organization and delivery (eg, how services were delivered: remote *versus* face to face; the number of patients accessing the service) and the creation of new services (eg, *ad hoc* created digital technologies and TMH) to overcome the challenges related to the COVID-19 pandemic. Documents solely reporting diagnostic aspects (eg, differential rates of two or more subgroups of mental health diagnoses accessing the service and frequency of mental health symptoms during the pandemic) were excluded, as these data describing part of a service do not provide information about the overall level of activity of a service. We included all types of primary research reports (randomized studies, observational studies, case reports, etc) focusing on mental health services and coronaviruses. No time or language restriction was applied.

### Data Extraction

Data extraction was performed independently by at least two reviewers (CZ, EGO, GR, JSWH, KR, KAS, OM, or RS). Relevant data on country of origin, data collection period, service delivery method (ie, face-to-face or TMH), and service provision were extracted from the included papers. We categorized the identified records according to the following categories: randomized controlled trials, cohort studies, before-and-after studies, case-control studies, case reports/case series, and survey/audit. For each paper, we also extracted the key quantitative findings and collected them in a descriptive summary.

### COVID-19 Stringency Index

Given that several waves of infections and consequent restrictions related to the COVID-19 pandemic affected different countries at different times, we considered the timing of the study period of the individual studies to be of limited use in contextualizing findings locally. Instead, we reported findings in the context of the country-specific COVID-19 Stringency Index, a composite measure developed within the Our World in Data project by the Global Change Data Lab (a nonprofit organization) and the University of Oxford [20] to reflect the stringency of a government’s response to COVID-19 restrictions at any specific time. The score is based on 9 response indicators, including school closures, workplace closures, and travel bans, rescaled to a value from 0 to 100 (where 100 is the strictest measure of response). This is therefore a dynamic measure, capturing changes in a country’s policies at any point throughout the pandemic. Where policies vary within a particular country, the index reflects the response level of the strictest subregion [11]. For each individual study, we report the minimum and maximum Stringency Index score for the relevant time period. The country-specific graphical representation of the COVID-19 Stringency Index starting from January 2020 can be accessed online [20].

## Results

### Overview

We identified a total of 24,339 records, and the number reduced by 3699 records after the removal of duplicates. After the abstract and full-text screening, an additional 20,539 records were excluded (Multimedia Appendix 2). A total of 101 papers were therefore included in the review. Of these, 60 were before-and-after studies, 23 were case reports and case series, and 18 were cross-sectional surveys. A summary of the 101 included articles is reported in Multimedia Appendix 3. Overall, the studies were concentrated in high-income countries, with the majority in Europe (39.6%) and North America (29.7%) (Multimedia Appendix 4).

### Face-to-Face Mental Health Services

Of the 101 articles, 72 reported data on face-to-face mental health services. A total of 52 of these were before-and-after studies, 7 were case reports and case series, and 14 were cross-sectional surveys. Regionally, 31 studies reported data from Europe, 20 from North America, 1 from South America, 11 from Asia, and 9 from Australia (Multimedia Appendix 5). No studies reported quantitative data from low-income countries, and none were from the African subcontinent. As shown in Multimedia Appendix 3, several studies reported a reduction in face-to-face visits [21,22] and emergency department presentations [23], with a reduction in the activity of specialized settings, such as electroconvulsive therapy clinics [24-27]. Data on hospitalizations were less clear, with some studies reporting evidence of a reduced number of admissions (eg, [28,29]), others reporting evidence of a rapid increase in admissions soon after the pandemic onset (eg, [23,30]), and still others reporting no differences compared to the same period in the previous year (eg, [31,32]).

### TMH Services

Table 1 [33-84] summarizes the characteristics of the subgroup of 55 articles reporting data on TMH services, which includes 23 before-after studies, 19 case reports or case series, and 13 cross-sectional surveys. Most of the collected data within these primary sources refer to the timeframe between January and October 2020, with most focusing on the period around March 2020.

**Figure 1:**
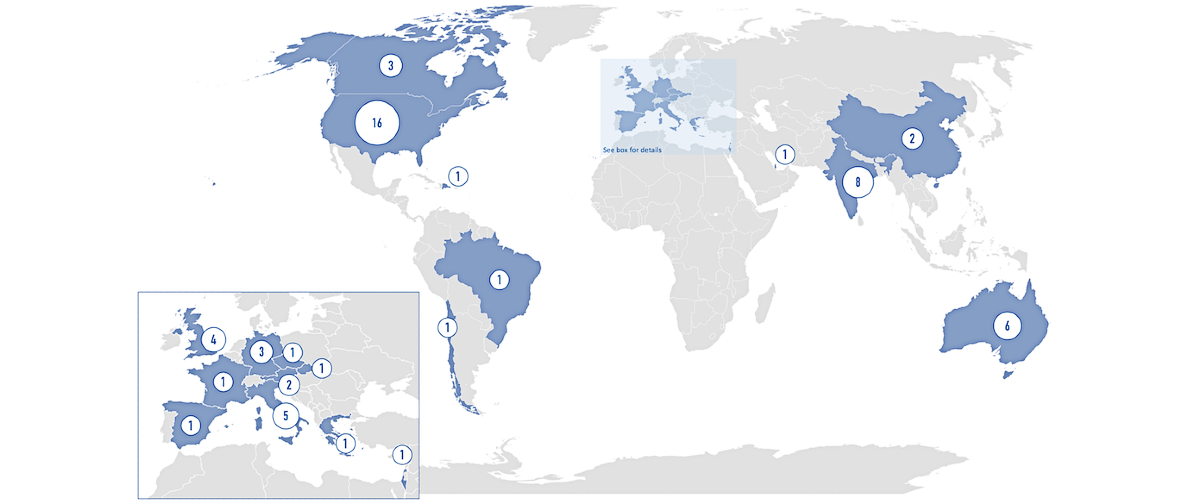
The distribution of articles reporting data on telemental health. Values in the circles indicate the number of articles.

The world distribution of the studies on TMH services (Figure 1) showed a higher representation in North America (n=18) and Europe (n=16, including 19 countries), while fewer articles reported data from Central or South America, Asia, and Australia. No studies reported quantitative data from low-income countries, and none were from the African subcontinent.

**Table 1:** Characteristics of the included studies on telemental health (N=55).

Study ID (authors, year)	Study design	Study setting	Country	Study period			Stringency Index, value or range	
				Start date	End date			
Arendt et al, 2020 [33]	Before-after study	Crisis hotline services for the general population	Germany and Austria	January 1, 2020	April 20, 2020	0.00-81.48		
Beran et al, 2020 [34]	Before-after study	A large, academic, consultation-liaison service	United States	January 2, 2020	June 6, 2020	5.56-72.69		
Berthaut and Chamignon, 2021 [35]	Descriptive study	A health care and education center for adolescents	France	March 17, 2020	May 18, 2020	76.85-87.96		
Cantini et al, 2020 [36]	Descriptive study	A psycho-oncology unit	Italy	March 3, 2020	June 3, 2020	67.59-93.52		
Carpiniello et al, 2020 [37]	Survey	71 departments of mental health and 107 psychiatric wards in general hospitals	Italy	April 1, 2020	April 11, 2020	85.19-87.96		
Connoly et al, 2020 [38]	Before-after study	The Department of Veterans Affairs	United States	March 11, 2020	April 22, 2020	21.76-72.69		
Datta et al, 2020 [39]	Before-after study	An eating disorder multidisciplinary unit	United States	March 1, 2020	May 31, 2020	8.33-72.69		
Garcia-Huidobro et al, 2020 [40]	Before-after study	A large private academic health network	Chile	March 1, 2020	April 30, 2020	0.00-73.15		
Ghiretti et al, 2020 [41]	Descriptive study	A free telephone-based psychological support service	Italy	March 18, 2020	June 3, 2020	67.59-93.52		
Ghosh et al, 2021 [42]	Before-after study	An outpatient service for substance use disorder	India	May 18, 2020	August 31, 2020	81.94-87.50		
Graell et al, 2020 [43]	Before-after study	A hospital outpatient service	Spain	March 16, 2020	May 10, 2020	68.98-85.19		
Grover et al, 2020 [26]	Survey	109 private and public mental health services	India	May 1, 2020	May 18, 2020	81.94-96.30		
Grover et al, 2020 [27]	Survey	396 private mental health services	India	May 1, 2020	May 15, 2020	81.94-96.30		
Guinart et al, 2021 [44]	Survey	818 mental health care professionals	United States	April 1, 2020	May 31, 2020	72.69		
Hall and Sukhera, 2020 [45]	Descriptive study	A virtual emergency psychiatric consultation service	Canada	Unknown	Unknown	Unknown		
Hames et al, 2020 [46]	Survey	93 clinic directors or designated representatives of the Association of Psychology Training Clinics	United States	March 11, 2020	March 31, 2020	21.76-72.69		
Harrison et al, 2020 [47]	Descriptive study	A telephone-based program to address substance abuse within emergency departments	Canada and United States	Unknown	Unknown	Unknown		
Hazarika et al, 2021 [48]	Survey	A psychological helpline for the general population	India	April 7, 2020	April 24, 2020	96.30-100.00		
Hoffnung et al, 2021 [49]	Before-after study	A community behavioral health center	United States	January 1, 2020	June 30, 2020	0.00-72.69		
Humer et al, 2020 [50]	Before-after study	338 health care professionals	Germany, Czech Republic, and Slovakia	March 24, 2020	May 20, 2020	54.63-87.04		
Johnson et al, 2020 [51]	Survey	2180 mental health care workers	United Kingdom	January 1, 2020	May 1, 2020	0.00-72.69		
Karim et al, 2020 [52]	Before-after study	Mental health outpatient services in Qatar	Qatar	March 1, 2020	June 30, 2020	13.89-86.11		
Khanra et al, 2021 [53]	Descriptive study	A large psychiatric hospital	India	April 1, 2020	September 30, 2020	81.94-100.00		
Lian et al, 2020 [54]	Before-after study	A hospital-based psychological counseling program	China	January 23, 2020	June 30, 2020	44.91-81.94		
Looi et al, 2020 [55]	Before-after study	Private psychiatric consultation services in Australia	Australia	April 1, 2020	June 30, 2020	62.04-73.15		
Looi et al, 2020 [56]	Before-after study	Psychiatric consultation services in Australian rural settings (Australian Capital Territory, Northern Territory, South Australia, and Tasmania)	Australia	April 1, 2020	May 31, 2020	64.35-73.15		
Looi et al, 2020 [57]	Before-after study	Private psychiatric consultation services in New South Wales, Queensland, Victoria, and Western Australia regions	Australia	April 1, 2020	May 31, 2020	64.35-73.15		
Looi et al, 2021 [58]	Before-after study	Private psychiatric consultation services in Australia	Australia	July 1, 2020	September 30, 2020	68.06-75.46		
Lunsky et al, 2021 [59]	Survey	942 direct support professionals working with people with intellectual and development disabilities	Canada	July 2, 2020	August 10, 2020	67.13-68.98		
Mehtani et al, 2021 [60]	Descriptive study	A telephone-based program for people with substance use disorders staying at San Francisco’s COVID-19 isolation and quarantine site	United States	April 10, 2020	May 25, 2020	72.69		
Mishkind et al, 2020 [61]	Descriptive study	An outpatient clinic	United States	March 2, 2020	April 10, 2020	11.11-72.69		
Moreland et al, 2021 [62]	Before-after study	An outpatient service for mental health and substance use treatment to pregnant and postpartum women	United States	March 1, 2020	May 31, 2020	8.33-72.69		
Myers Virtue et al, 2021 [63]	Descriptive study	A psychosocial oncology service	United States	March 1, 2020	September 30, 2020	8.33-72.69		
Naik et al, 2021 [64]	Descriptive study	An outpatient service	India	March 25, 2020	May 31, 2020	81.94-100.00		
Peppou et al, 2020 [65]	Descriptive study	A helpline for the general population	Greece	March 22, 2020	April 13, 2020	74.04-84.26		
Peralta et al, 2020 [66]	Descriptive study	A counseling service for the general population	Dominican Republic	March 25, 2020	May 17, 2020	92.59-100.00		
Perricone et al, 2021 [67]	Descriptive study	A national psychological counseling service	Italy	April 1, 2020	June 30, 2020	67.59-93.52		
Pierce et al, 2020 [68]	Survey	2619 psychologists	United States	May 11, 2020	May 25, 2020	72.69		
Prior, 2020 [69]	Descriptive study	A psycho-oncology unit	Italy	Unknown	Unknown	Unknown		
Probst et al, 2020 [70]	Survey	1547 psychotherapist	Austria	March 24, 2020	April 1, 2020	81.48		
Rainwater et al, 2020 [71]	Before-after study	An outpatient service for consultation and counseling of cancer patients	United States	April 1, 2020	September 30, 2020	62.50-72.69		
Ravindran et al, 2020 [72]	Descriptive study	A national helpline for the general population	India	Unknown	Unknown	Unknown		
Rosen et al, 2020 [73]	Before-after study	The Veterans Health Administration – the largest integrated service in the United States	United States	January 1, 2020	June 30, 2020	0.00-72.69		
Salum et al, 2020 [74]	Descriptive study	A community mental health service	Brazil	March 23, 2020	March 23, 2020	71.76		
Sampaio et al, 2021 [75]	Survey	768 mental health professionals	United States	April 24, 2020	May 18, 2020	72.69		
Sharma et al, 2020 [76]	Descriptive study	A large child psychiatry department	United States	February 28, 2020	April 3, 2020	5.56-72.69		
Singh Bhandari, 2020 [77]	Descriptive study	An outpatient clinic	India	March 25, 2020	May 26, 2020	81.94-100.00		
Staples et al, 2020 [78]	Before-after study	A national digital mental health service for people experiencing anxiety and depression	Australia	March 19, 2020	June 10, 2020	44.44-73.15		
Steeg et al, 2021 [79]	Before-after study	General practice services in England	United Kingdom	March 10, 2020	June 10, 2020	11.11-79.63		
Stewart and Broadbent, 2020 [22]	Before-after study	A large mental health service	United Kingdom	February 16, 2020	April 16, 2020	11.11-79.63		
Stewart et al, 2020 [80]	Before-after study	A large mental health service	United Kingdom	February 1, 2020	May 15, 2020	11.11-79.63		
Vonderlin et al, 2021 [81]	Descriptive study	A hotline for psychological first aid for the general population	Germany	April 22, 2020	July 24, 2020	55.09-76.85		
Yaffa et al, 2021 [82]	Before-after study	An eating disorder treatment center for adolescents	Israel	January 1, 2020	October 31, 2020	0.00-94.4		
Zhong et al, 2020 [83]	Survey	108 Chinese residents	China	January 27, 2020	February 2, 2020	69.91-77.31		
Zulfic et al, 2020 [84]	Survey	A community outpatient service	Australia	Unknown	Unknown	Unknown		

The key findings of included papers involving the impact of COVID-19 on the use of TMH are shown in Textbox 1. Overall, studies set in 19 countries reported the implementation of TMH services locally at the beginning of the pandemic. They include inpatient and outpatient services (eg, [71]), specialized services (eg, [38,82]), adolescent services (eg, [82]), and services in both private and public sectors (eg, [27]). For those services in which TMH was already available, an increase in its use during the pandemic was reported in several countries (eg, [21,50,70]). The specific COVID-19 Stringency Index chart for each country throughout the pandemic can be accessed online [20].

Several studies reported the creation of helplines both locally and within national programs in several countries to provide psychiatric and psychological help to the general population (eg, [48,65,67,72]).

**Telemental health services around the world. The specific COVID-19 Stringency Index chart for each country can be accessed online [20].**

**Asia**

***China***

*Before-after study*

• A newly created hospital-based psychological counseling program in China served 474 users (7 users per day) during the lockdown, and the number reduced to 162 (2 users per day) after April 2020 [54].

*Survey/audit*

• 42.6% of 108 survey respondents who received a psychiatric consultation during lockdown attended an internet-based service (n=46), 37.0% attended a face-to-face visit (n=40), and 20.4% attended a telephone-based service (n=22) [83].

***India***

*Before-after study*

• In India, a telemedicine-assisted stepped-care outpatient service for substance use disorder was developed and started on the May 18, 2020. By August 31, 2020, 160 new and 219 follow-up patients were registered in the teleaddiction service. Among them, 128 (80%) and 198 (90.2%) patients received teleconsultations, respectively [42].

• Compared to the same period of 2019, the number of patients seen in a psychiatric outpatient service for patients with substance abuse dropped (May: 170 vs 22; June: 351 vs 43; July: 467 vs 44; August: 436 vs 51) [42].

*Descriptive study*

• A local newly created psychological helpline received 239 calls over the 18 days of lockdown (April 7-24, 2020) [48].

• The Indian Government helpline received 20,475 calls during the first month of activity [72].

• An outpatient service in India created a new telephone outpatient service and delivered 60.1% of the planned follow-up visits using telehealth (1049 of 1748) in the first 2 months of lockdown [64].

• During the first months of the lockdown, a team of psychiatrists of an Indian hospital made 78 teleconsultations with new and followed-up patients. In 6 cases, the patients were referred to their local hospital for further face-to-face assessment [77].

• In a large psychiatric hospital in India, 168 consultations were made using telepsychiatry between April and September 2020. The number of teleconsultations during lockdown was positively correlated with travel cost savings (ρ=0.47, *P*<.01) and time savings (ρ=0.49, *P*<.01) [53].

*Survey/audit*

• A survey comparing the services in both public and private institutes during the lockdown period in India (n=109) showed that telecommunication service use increased from 19.3% to 45.9% comparing the period before and during the pandemic [26].

• A survey evaluating the services in the private sector provided during the lockdown period in India (n=396) showed that telecommunication service use increased from 26.3% to 52.0% comparing the period before and during the pandemic [27].

***Israel***

*Before-after study*

• In an eating disorder treatment center for adolescents, telemedicine meetings comprised 37% of all sessions during January to October 2020 (2193 sessions), while they were not used during the respective period between 2015 and 2019 [82].

***Qatar***

*Before-after study*

• Analyzing data on the number of visits in the mental health outpatient setting in Qatar in the 4 months following the introduction of telepsychiatry (March-June 2020), the total number of individuals accessing mental health services (both face-to-face and telepsychiatry) increased by approximately 36.5% compared to the same period in 2019 (22,086 and 16,175 cases, respectively) [52].

**Australia**

***Australia***

*Before-after study*

• The use of the MindSpot Clinic, a national digital mental health service (DMHS) providing services to people experiencing anxiety and depression, increased by 16.7% (mean number of assessments per week=455) during the pandemic (March 19 to June 10, 2020) compared to the period between September 1 and September 28, 2019 (390 assessments per week) [78].

• Comparing data on private psychiatrists’ visits held in April-June 2020 and the same period in 2019 in Australia, the number of psychiatry consultations (telehealth and face-to-face) rose during the pandemic by 14%, with telehealth representing nearly half of this total. Face-to-face consultations in 2020 were only 56% of the comparative number of 2019 consultations. Most telehealth involved short telephone consultations of ≤15-30 min. Video consultations comprised 38% of total telehealth provision [55].

• Comparing data on visits held in Australian rural settings (Australian Capital Territory [ACT], Northern Territory [NT], South Australia [SA], and Tasmania [TAS]), the overall rate of consultations (face-to-face and telehealth) increased during March and April 2020, compared to the monthly face-to-face consultation average of July 2018 to June 2019, except TAS (ACT: 1724 in April, increased 114%; 2061 in May, increased 136%; NT: 296 in April, increased 108%; 337 in May, increased 123%; SA: 12,864 in April, increased 116%; 12,876 in May, increased 116%; TAS: 1886 in April, reduced 85%; 2189 in May, reduced 98%). For total video and telephone telehealth consultations combined, video consultations were lower in April 2020 and higher in May 2020 [56].

• The total combined use of telehealth and face-to-face private psychiatric consultation services in New South Wales, Queensland, Victoria, and Western Australia regions (Australia) in April and May 2020 increased by 10%-20% of the average monthly face-to-face consultations in the 2018/19 financial year [57].

• Comparing data on private psychiatrists’ visits held in July-September 2020 and the same period of 2019 in Australia, the number of psychiatry consultations (telehealth and face-to-face) rose during the pandemic by 14%, with telehealth representing 43% of this total. Face-to-face consultations in 2020 were only 64% of the comparative number of 2019 consultations. Most telehealth involved short telephone consultations of ≤15-30 min. Video consultations comprised 42% of total telehealth provision [58].

*Survey/audit*

• An audit of 314 community patients to examine the potential implications of telephone support found that 21 (7%) did not have access to a phone, and a further 58 (18%) were reported by the original authors as “unreliable in responding to contact over the phone based on past clinician experience.” Moreover, during the first wave, regular face-to-face reviews were necessary for a group of patients, including 91 patients (29%) treated with depot medications, and 71 (23%) taking clozapine [84].

**Europe**

***Austria***

*Survey/audit*

• During the lockdown, the number of patients treated daily via telephone increased from 0.42 ± 3.01 to 4.53 ± 5.77 (+979%, *P*<.001), and via internet from 0.18 ± 1.35 to 2.99 ± 4.44 (+1561%, *P*<.001) [70].

***Austria and Germany***

*Before-after study*

• The number of crisis hotline calls increased during lockdown in both Austria and Germany [31].

***Czech Republic, Germany, and Slovakia***

*Survey/audit*

• A survey on 338 health care professionals showed that the number of patients treated via telephone increased from 0.92 ± 3.16 to 3.28 ± 5.22 per week (+257%, t −8.717, *P*<.001), and the number of patients treated via the internet increased from 0.59 ± 2.54 to 5.83 ± 6.82 per week (+888%, t −15.346, *P*<.001) during the 2-month period of confinement [50].

***France***

*Descriptive study*

• During lockdown, a health care and education center for adolescents that provides long-term psychiatric care, created a virtual ward using Discord, a platform used to communicate using chat, calls, and video calls, to ensure continuity of care. Of the 38 patients who used this service, only 6 withdrew early [33].

***Germany***

*Descriptive study*

• A total of 1292 telephone consultations were made by a telephone hotline for psychological first aid for COVID-19–related burden in 4 months covering a period during and after the German lockdown. In 42% of all consultations, a short therapeutic intervention was performed; in 26%, psychotherapeutic treatment was recommended; and in 11%, referral to other specialized telephone services was made [81].

***Greece***

*Descriptive study*

• In a report from a mental health helpline service in Greece during lockdown, most calls pertained to the quarantine (n=482, 83.7%) and more specifically to feelings of “restraint” (56%) and “loneliness” (53%) [65].

***Italy***

*Descriptive study*

• In Emilia Romagna region (Italy), a free telephone-based psychological support service was established during lockdown, receiving 312 calls in the first 11 weeks of activity [41].

• A national telephone psychological counseling service established during the COVID-19 pandemic had 193 users between April and June 2020 [67].

• A psycho-oncology unit reported having treated 28 cancer patients, 9 caregivers, and 7 family members during April-September 2020, switching from face-to-face visits to virtual consultations [36].

• In Treviso (Italy), the oncology unit switched their psychological monitoring to a virtual modality during the pandemic. In the first 9 weeks, they conducted 123 online visits (12% were video calls using Google Hangouts, and 88% were telephone calls) [69].

*Survey/audit*

• Data from 71 departments of mental health and 107 psychiatric wards in general hospitals showed that during the lockdown (April 2020), scheduled psychiatric consultations, both at home and on-site, went ahead for selected cases, being replaced in approximately 75% of cases by scheduled remote contact, mainly telephone calls (100%), video calls (67%), or emails (19%), with 41% of units adopting all these means of contact [37].

***Spain***

*Before-after study*

• During the early COVID-19 pandemic (March-May 2020) in a hospital in Madrid, out of 1818 outpatient consultations carried out, 1329 (73.10%) were delivered by telephone or videoconferencing and 489 (26.9%) were face-to-face, corresponding to 365 patients who were receiving treatment at the time in the outpatient clinic or day hospital [43].

***United Kingdom***

*Before-after study*

• The likelihood of receiving a remote general practitioner/practice nurse consultation within 3 months of a self-harm episode was higher in the COVID-19 pandemic (67.7%; March-April 2020) than in the prepandemic period (32.3%; ratio 2.10, CI 2.05-2.15; same period of 2010-2019). The overall likelihood of having a general practitioner/practice nurse consultation was slightly lower (80.3% vs 83.2%; ratio 0.97, CI 0.96-0.98) [69].

• Comparing the 31-day periods before and after March 16, 2020 (lockdown announcement), virtual contacts increase by 117% compared to a 3%-22% reduction observed comparing the same periods between 2015 and 2019 [25].

• Comparing the period before March 16 to that between March 16 and May 15, 2020, virtual contacts with community mental health teams increased from 154 ± 17 to 380 ± 97 (+147%). In the same period, mean virtual contacts increased by 102.7% (26.7 ± 7.3 vs 54.2 ± 14.8) and mean total contacts reduced by 24.9% (161.9 ± 30.7 vs 121.5 ± 26.1). Daily caseloads reduced by 2.1% (8729 ± 24 vs 8539 ± 124) and by 26.4% (221.8 ± 8.5 vs 163.3 ± 20.0) [80].

*Survey/audit*

• During the first COVID-19 wave, 61.1% of 2180 mental health care workers in the United Kingdom rated the adoption of new digital ways of working as “very or extremely important” in the management of the impact of COVID-19 at work [51].

**North America**

***Canada***

*Descriptive study*

• In Ontario, the implementation of a virtual emergency psychiatric consultation service (ie, Emergency Diversion Clinic) during the pandemic allowed provision of consultations for 60% of all youth presenting to the emergency department for a mental health issue. After the assessment, 56% of patients were linked to community mental health support [45].

*Survey/audit*

• A Canadian survey of direct support professionals (n=942) working during the pandemic with people with intellectual and development disabilities, showed that the majority of them completed telephone-based visits (n=549, 58%), and only 22% (n=204) made at least one videocall. A total of 225 (24%) responders attended at least one face-to-face visit [59].

***Canada and United States***

*Survey/audit*

• In March 2020, an online survey involving 93 clinic directors or designated representatives of the Association of Psychology Training Clinics mainly in the United States (n=89) and Canada showed that 23.7% (n=22) were forced to close face-to-face clinics and discontinue services, at least temporarily. Of those that remained open for services, 61 training clinics (65.6% of the total sample, 86% of those that remained open) reported that their sites remained open primarily using telepsychology [46].

***United States***

*Before-after study*

• Psychiatric consultations and patient volume in North Carolina decreased with the onset of the pandemic (March 2020) by 66.9% and 25.2%, respectively. After the introduction of video consultations, psychiatric consultations were 39.1% lower compared to prepandemic data [34].

• Daily telemental health visits to the Department of Veterans Affairs rose from 1739 to 11,406 (+556%) in the weeks following the pandemic declaration (March 2020). Daily in-person encounters fell from 57,296 to 10,931 (−81%) [38].

• In an American eating disorder multidisciplinary unit, the yearly average inpatient census by month changed from 31.92 (SD 5.33; before the COVID-19 pandemic) to 19.33 (SD 4.5; March 2020 to May 2020) (59% of the usual census), owing to the implementation of telehealth strategies in most aspects of the services (eg, new admission evaluation, psychotherapy, and group therapy) [39].

• After the onset of COVID-19 (April-May 2020), the average monthly utilization of telehealth services within the Medical University of South Carolina’s Women’s Reproductive Behavioral Health Program (which provides outpatient mental health and substance use treatment to pregnant and postpartum women within obstetric practices) increased by 90% compared to the data prior to the pandemic [62].

• Comparing delivery of telemental health services in New York (USA) before, during, and after the lockdown, there was a tendency for adult patients (n=1115, 21,131 sessions) to prefer telehealth compared to children (n=1374, 22,163 sessions) (*P*<.001). In this service, telehealth was implemented in March 2020 (onset of lockdown) but patients returned to prefer face-to-face visits when in-person services resumed in May and June 2020 (χ^2^=21.745, *P*<.01) [49].

• Data were compared for the number of mental health consultation visits made by cancer patients face-to-face in April-September 2019 and both face-to-face and virtually in April-September 2020. In 2020, the number of inpatient consultations (330 vs 623), referrals (127 vs 175), and outpatient visits (448 vs 550) decreased. Of the outpatient visits, 359 were telephone contacts, 69 were video visits, and only 20 were in person [71].

• Data from the Veterans Health Administration (VHA), the largest integrated health care system in the United States, showed that before the pandemic (October 2019 to February 2020), VHA had 1.5 to 1.8 million visits per month, of which 85% were in-person visits, 11% were by telephone, and 5% were by video. The use of telepsychiatry increased with the pandemic, and in June 2020, there were 1.5 million visits, of which 19% were in person, 59% were by telephone, and 20% were by video [73].

*Descriptive study*

• During March 2020, a large child psychiatry department registered an overall reduced number of study visits (645 vs 358) over a 1-month period, with a shift toward phone and home-based telemental health (n=171 and n=160, respectively), compared to in-person visits (n=27) [76].

• In the 2 weeks before the implementation of the “Addiction Telehealth Program” (ATP) in April 2020, which is a telephone-based program to reduce treatment access barriers for people with substance use disorders staying at San Francisco’s COVID-19 isolation and quarantine site, the program received 10 calls from other health care providers, whereas in the 6 weeks after the implementation (April 10-May 25, 2020), there were 59 consultations for the isolation and quarantine site [60].

• Data from the Johnson Depression Center and the Steven A. Cohen Military Family Clinic at the University of Colorado Anschutz Medical Campus showed that the change to telemental health helped reduce no-show rates from 11.4% (n=57) in the 2 weeks before the pandemic (March 2-13, 2020) to 7.8% (n=38) after implementation of telehealth (March 30-April 10, 2020). In the following months (April-September 2020), the no-show rate was stable, with a rate between 5.5% and 8.5%, and a 26.2% increase in overall completed visits [61].

• During the first wave (March-September 2020), a psychosocial oncology service in the United States continued to provide visits through telepsychology for 93% of established patients (n=85), while 4 patients left the service and 2 patients preferred to wait for the reintroduction of face-to-face visits. The service also received 263 new referrals and delivered a first visit for 50.6% (n=133), with 82% (n=109) as digital visits and 18% (n=24) as face-to-face visits [63].

• In New York, a telephone-based model was implemented during the pandemic to continue to address substance use within emergency departments (SBIRT program). In 13 weeks, there were 228 incoming calls, 190 outgoing calls, and 4 voicemails processed. Moreover, 108 (26%) calls were with patients, 13 (3%) with family/friends, 224 (53%) with staff members, and 79 (19%) with treatment providers [47].

*Survey/audit*

• Of the 818 mental health care professionals in the United States who reported using telepsychiatry during the pandemic (April-May 2020), 500 (61%) used both video and telephone, 273 (33%) used only telephone, and 45 (6%) used only video. Among the advantages cited, flexible scheduling or rescheduling, timely appointment starts, and lack or reduction of no-shows were reported. Among the raised concerns, there were technical difficulties and difficult access to video platforms, forcing the provider and patient to conduct visits telephonically despite both parties preferring 2-way video [44].

• A survey of psychologists showed that the use of telepsychology increased with the pandemic, compared to data prior to the pandemic (before January 2020), from 7.07% (SD 14.86) to 85.53% (SD 29.24) of the total activity [68].

• In a survey about the use of telepsychology, data showed that most therapists who already used telepsychology before COVID-19 reported an overall increase in requests for therapy services from current patients (36.5%), whereas 41% of those who had started to use telepsychology during the pandemic reported an overall decrease. Both groups reported a decrease in the number of requests from new clients (45% and 53%, respectively) [75].

**Central and South America**

***Brazil***

*Descriptive study*

• In a community mental health service in Brazil, telephone contact was possible for 61% of 154 treated patients, with 29% being advised face-to-face visits through regular service attendance, and around 7% of service users were unable to be contacted, despite several attempts [74].

***Chile***

*Before-after study*

• During the early pandemic, a large private academic health network implemented a new telemental health service. In Santiago, the number of visits delivered during this period via telepsychiatry was comparable to the 24.7% of visits delivered in 2019 by the face-to-face service [40].

***Dominican Republic***

*Descriptive study*

• In Santo Domingo, a team of volunteers consisting of 598 psychologists and 70 psychiatrists provided telephone counseling during the pandemic. In the period from March 25 to May 17, 2020, they conducted 6800 phone interventions [66].

## Discussion

### Principal Findings

Our systematic review found that face-to-face delivery of mental health services was reduced at the start of the pandemic period, with a reduction in the total number of delivered visits and few mental health services forced to close. To meet mental health needs, the use of TMH rapidly increased across high-income countries and low- and middle-income countries at the beginning of the pandemic, with almost complete virtualization of many services by the end of the first year. To the best of our knowledge, this is the first review that, using a preplanned systematic approach, has collected quantitative reports on the extent to which the COVID-19 pandemic impacted mental health services internationally. Moreover, in presenting findings within the context of the relevant COVID-19 Stringency Index, service changes can be understood in the context of how strict national restrictions were throughout the pandemic.

At the beginning of the pandemic, TMH was increasingly adopted by mental health services to overcome the challenges related to COVID-19 restrictions, with a steep rise during the confinement period and, in general, during periods when the COVID-19 Stringency Index was higher. In some settings, this implementation was followed by a greater number of total health care consultations than in previous years, when the only option was face-to-face visits (eg, [34,52,55-58]). Moreover, it also involved specific and specialist (tertiary) mental health service provisions, such as emergency department or services dedicated to postpartum women and patients with cancer. This adaptation was essential to continue following up patients who may have been considered at high risk or as a vulnerable category during the pandemic due to their specific conditions.

The transition to the use of TMH was reported across high- and middle-income countries, but there were no studies from low-income countries. TMH provision has great potential in low-and middle-income countries due to large geographical distances and limited availability of mental health services to cover the population needs [85], combined with reduced health system capacity during the COVID-19 pandemic [86]. However, very few studies quantitively assessed the impact of the COVID-19 pandemic in middle-income countries (Iran, Turkey, Dominican Republic, India, China, and Brazil) and none assessed the impact specifically in low-income or African subcontinent countries. This is despite reports of the rollout of TMH in African nations to provide quicker and more extensive access to mental health services [87]. Additionally, models of service transformation in high-income countries may be difficult to replicate or be less relevant in these settings [88]. Clearly, quantitative data are needed to understand the impact of the COVID-19 pandemic on mental health services and longer-term changes, both positive and negative, in low- and middle-income countries [89].

Although some services provided evidence of a gradual shift back to face-to-face contact with a reduction in the COVID-19 Stringency Index (eg, after the initial lockdown period), virtual delivery methods continued to be used more than in the prepandemic period and, in some cases, remained the method for the majority of patients. This may not have been universal across all services and may have varied with patient preferences and specific characteristics. For example, a US study found that telehealth was less preferred for children after the confinement period compared to adults due to difficulties delivering therapy to them remotely [49]. Moreover, findings indicated that TMH was favored for psychiatric consultations and support services, but less for psychotherapy in both adult and child services [49]. This was despite the findings of a recent systematic review that videoconferencing was an accessible and feasible modality for therapy delivery to adult patients, with comparable levels of therapeutic alliance between virtual and in‐person therapy [90]. This apparent discrepancy might be due to the challenging settings that clinicians and patients faced using the newly created TMH services during the pandemic, but it also suggests that the problems might be remedied by providing training (for both patients and clinicians) in TMH to improve delivery of therapies [15]. Clinicians’ attitudes toward TMH may also have an impact on its implementation in daily practice. As shown in a recent systematic review on barriers to and facilitators of TMH during the pandemic, clinicians reported a reduction in their ability to develop and maintain a therapeutic relationship during televisits [91]. This aligns with prepandemic data that showed that clinicians’ perspectives on TMH can be a major barrier to the uptake of the virtual modality [92]. Other factors influencing clinicians’ adoption of TMH include perceptions of how effectively this can be delivered, fears that aspects of in-person care may be missed in virtual encounters (eg, observation of physical signs such as tremor, fidgeting, and anxiety), and satisfaction with plans for handling clinical emergencies at a distance [92,93]. Moreover, a survey conducted in Hong Kong during the pandemic highlighted the need for mental health staff to receive dedicated training on clinical, technological, and program-specific aspects when providing TMH [94]. There is well-established guidance on virtual consultations to support health care professionals in the pandemic [95-97]. This includes physical examination, risk assessment, and management of emergency situations such as agitation/aggression, suicidality, and domestic violence [98]. During the COVID-19 pandemic, charities and other organizations reported an increase in cases of domestic violence and provided guidance on the safe assessment of domestic abuse during virtual contacts [99]. Although these recommendations may have been helpful to guide staff through the first period of the pandemic, training for clinicians will be crucial to take advantage of TMH in standard clinical care beyond the pandemic [15]. For example, implementing telehealth as part of the formal training for psychiatric residents has been proposed as an effective means of developing these skills [100].

While TMH was implemented for most patients, there were a small number of specific groups where it was not possible to be used alone, and instead, a hybrid model was used, combining face-to-face and digital strategies. This included services providing treatment with clozapine or depot medication, and electroconvulsive therapy. During the pandemic, such services applied several strategies to reduce the risk of exposure for these patients (eg, increasing the timing between blood monitoring for clozapine, new infection control measures, and social distancing) [101], but face-to-face contact could not be avoided completely. This highlighted that while teleassessment may have similar advantages and disadvantages independently to patient diagnoses, implementation of telemedicine in treatment management will face different barriers in different diagnostic groups. For instance, telemedicine has been typically used for the treatment of some diagnoses, such as anxiety and eating disorders [102,103], while programs addressing other diagnostic groups are now emerging [104].

Due to financial barriers, digital literacy, and features of chronic mental illness, a reliance on TMH services is not feasible for a minority of community patients [84]. Studies describing these clinical groups report a higher prevalence of certain diagnoses (eg, schizophrenia or schizoaffective disorder) [84], while others indicated several barriers, such as lack of digital literacy or confidence to make full use of a telephone, and lack of an appropriate device or broadband required to connect with clinicians [105]. In resource-limited settings during the COVID-19 pandemic, mental health staff working for Médecins Sans Frontières used audio-only platforms for 80% of TMH care interventions [106]. Despite this, over half of the patients were unreachable using these interventions, mainly due to poor network coverage, lack of communication devices, or lack of a private space at home [106]. For these patients, specific provision of a device or broadband might be necessary. Investment in providing these resources will have to be balanced at a country level against the costs associated with the anticipated increase in mental health problems. Moreover, appropriate training in TMH as well as the use of digital tools specifically for patients may increase access to and acceptability of virtual means. For instance, the DOORS (Digital Opportunities for Outcomes in Recovery Services) program provides an example of training to increase digital literacy and confidence in patients with first-episode psychosis and serious mental illness [104]. Such models will need to be developed locally to match the needs of local populations and clinical groups.

### Limitations

Our study has some limitations. A systematic review relies on the quality of the primary papers, and in this case, although we found 55 articles on TMH, 19 were case reports or case series presenting descriptive data on local services, with limited generalizability. To address this, we grouped our findings according to the country of origin, considering differences in the local service structure, local needs, and stringency of confinement measures during the pandemic. In addition, we found limited reports on middle-income countries, and no reports from the African subcontinent or from low-income countries. Absent data do not represent evidence for the lack of effectiveness or feasibility, as services (such as those in African nations) may have implemented TMH without publishing quantitative data. The lack of representation of low- and middle-income countries in published health research is well documented [107], but without data, it is difficult to assess the impact of the pandemic on mental health services in these areas. Since the literature on COVID-19 is growing at a rapid pace, it is likely that relevant articles have been published after our final search and are therefore not included in our review. Finally, the aim of our project was to collect information about the changes and difficulties that mental health services faced at the start of the pandemic. Therefore, we limited the data collection to the first year of the pandemic. Future studies should evaluate whether these implementations will be confirmed or further refined.

### Conclusions

During sudden health emergencies or disasters, such as the COVID-19 pandemic, mental health services cannot rely on face-to-face activities to provide care to all patients. The implementation of TMH has been demonstrated as feasible and widespread in different countries, allowing mental health services to continue the provision of essential care for patients, even during times of extreme confinement. Although many high-income countries and some middle-income countries were able to transition mental health services to digital delivery, this brought some challenges. While TMH was predominantly used in the acute pandemic, some findings showed a reduction in its use with time. Further training and support will be needed to maintain the benefits of TMH and to address barriers, such as digital exclusion. The pandemic can be seen as an opportunity to explore the benefits of TMH in enabling broader access to high-quality psychiatric treatment in the longer term [108]. Telehealth delivery methods are not only useful tools for the acute pandemic, but also have a role in addressing the anticipated increase in mental health needs after the immediate disruption and in preparation for future emergencies [15]. However, specific subgroups of patients will likely need or prefer face-to-face care, while others may not have access to virtual means. A blended approach, combining in-person and virtual modalities, that takes into consideration the needs, preferences, and digital skills of patients may suit the future development of mental health services, but this requires further investigation that considers acceptability to patients, carers, and clinicians. Delivering effective and equitable blended approaches will require confidence in using digital technologies, training, and experience in all modalities [108,109] to ensure that the benefits of TMH and associated eHealth technologies spark a transformation that is sustained beyond the immediate crisis.

## Appendix

Multimedia Appendix 1 Search strategy.

Multimedia Appendix 2 PRISMA (Preferred Reporting Items for Systematic Reviews and Meta-Analyses) flowchart.

Multimedia Appendix 3 Impact of COVID-19 on mental health services: summary of key findings.

Multimedia Appendix 4 World distribution of 101 included papers.

Multimedia Appendix 5 Characteristics of 72 papers on face-to-face services.

## Abbreviations

MERS: Middle East respiratory syndrome
TMH: telemental health
WHO: World Health Organization
