# Supplementary material for: Impact of the COVID-19 Pandemic on the Global Delivery of Mental Health Services and Telemental Health: Systematic Review
Source: JMIR Ment Health. 2022 Aug 22;9(8):e38600. doi: 10.2196/38600 (PMC9400843; doi:10.2196/38600)
Supplement: Multimedia Appendix 1 [file mental_v9i8e38600_app1.docx]

**Multimedia Appendix 1.** Search strategy.

| **Pubmed**  (((((((((((((((((((((((((((((((((((((((((((((((((((((((((((((((((mental health) OR anorexi*) OR schizophreni*) OR psychos*) OR psychot*) OR psychiatr*) OR depress*) OR attempted suicide) OR suicid*) OR clozapine) OR lithium) OR bipolar) OR anxiety) OR National Early Warning Score) OR close observation) OR seclusion*) OR restraint*) OR (visit* AND (policy OR policies))) OR trauma) OR traumatic) OR violence) OR aggress*) OR agitat*) OR eating disorder*) OR bulimia) OR antipsychotic*) OR mood stabili*) OR antidepress*) OR "Health Services"[Mesh]) OR "Health Personnel"[Mesh]) OR self-harm) OR self-injury) OR NSSI) OR DSH) OR parasuicide) OR passive death wish) OR depot) OR rapid tranquil*) OR hypnotic) OR GABA-modulator) OR antimanic) OR ECT) OR TMS) OR psychotherapy) OR group therapy) OR CBT) OR cognitive behavioural) OR cognitive behavioral) OR dialectical) OR DBT) OR mentalisation) OR mentalization) OR MBT) OR sickness absence) OR occupational health) OR Balint group) OR mental health act) OR section) OR detain) OR community treatment order) OR section 17 leave) OR involuntary institutionalization) OR patient safety) OR "quality of care") AND ((((((((((coronavirus) OR COVID19) OR COVID-19) OR SARS-CoV-2) OR "COVID-19"[Supplementary Concept]) OR MERS) OR SARS) OR "SARS Virus"[Mesh]) OR "Middle East Respiratory Syndrome Coronavirus"[Mesh]) OR "Coronavirus"[Mesh]) NOT (animal[mh] NOT (human[mh))) AND English[Language]  **EMBASE**  ('mental health'/exp OR 'mental health' OR 'anorexi*' OR 'schizophreni*' OR 'psychos*' OR 'psychot*' OR 'depress*' OR ‘attempted suicide’ OR 'suicid*' OR 'clozapine'/exp OR 'clozapine' OR 'lithium'/exp OR 'lithium' OR 'bipolar' OR 'anxiety'/exp OR 'anxiety' OR 'national early warning score'/exp OR 'national early warning score' OR 'close observation' OR 'seclusion'/exp OR 'seclusion' OR 'restraint'/exp OR 'restraint' OR (('visiting'/exp OR 'visiting' OR 'visitor' OR 'visitors') AND ('policy'/exp OR 'policy' OR 'policies')) OR 'trauma'/exp OR 'trauma' OR 'traumatic' OR 'violence'/exp OR 'violence' OR 'aggress*' OR 'agitat*' OR 'eating disorder'/exp OR 'eating disorder' OR 'bulimia'/exp OR 'bulimia' OR 'psychiatry'/exp OR 'psychiatry' OR 'neuroleptic agent'/exp OR 'neuroleptic agent' OR 'antipsychotic*' OR 'antidepressant agent'/exp OR 'antidepressant agent' OR 'antidepressant*' OR 'mood stabili*' OR 'anxiolytic agent'/exp OR 'anxiolytic agent' OR 'benzodiazepine*' OR 'health care personnel'/exp OR 'health care personnel' OR 'health service'/exp OR 'health service' OR 'automutilation'/exp OR 'automutilation' OR 'self-harm'/exp OR 'self-harm' OR 'self-injury'/exp OR 'self-injury' OR 'nssi' OR 'dsh' OR 'suicide attempt'/exp OR 'suicide attempt' OR 'parasuicide'/exp OR 'parasuicide' OR 'passive death wish' OR 'depot'/exp OR 'depot' OR 'rapid tranquillisation' OR 'rapid tranquillization' OR 'hypnotic sedative agent'/exp OR 'hypnotic sedative agent' OR 'hypnotic' OR 'gaba-modulator' OR 'benzodiazepine receptor affecting agent'/exp OR 'benzodiazepine receptor affecting agent' OR 'antimanic' OR 'tranquilizer'/exp OR 'tranquilizer' OR 'ect'/exp OR 'ect' OR 'tms'/exp OR 'tms' OR 'psychotherapy'/exp OR 'psychotherapy' OR 'group therapy'/exp OR 'group therapy' OR 'cbt' OR 'cognitive behavioural therapy'/exp OR 'cognitive behavioural therapy' OR 'cognitive behavioural' OR 'cognitive behavioral' OR 'dialectical behavior therapy'/exp OR 'dialectical behavior therapy' OR 'dialectical' OR 'dbt' OR 'mentalisation'/exp OR 'mentalisation' OR 'mentalization'/exp OR 'mentalization' OR 'mbt' OR 'absenteeism'/exp OR 'absenteeism' OR 'sickness absence'/exp OR 'sickness absence' OR 'occupational health'/exp OR 'occupational health' OR 'balint group'/exp OR 'balint group' OR 'mental health act'/exp OR 'mental health act' OR 'section'/exp OR 'section' OR 'detain' OR 'community treatment order'/exp OR 'community treatment order' OR 'section 17 leave' OR 'involuntary institutionalization' OR 'patient safety'/exp OR 'patient safety' OR 'health care quality') AND ('coronavirus'/exp OR 'coronavirus' OR 'covid 19'/exp OR 'covid 19' OR 'sars coronavirus'/exp OR 'sars coronavirus' OR 'sars-related coronavirus'/exp OR 'sars-related coronavirus' OR 'mers' OR 'middle east respiratory syndrome coronavirus'/exp OR 'middle east respiratory syndrome coronavirus' OR 'sars'/exp OR 'sars' OR 'severe acute respiratory syndrome'/exp OR 'severe acute respiratory syndrome') AND [english]/lim  **COAP Living evidence on COVID-19 (formerly SARS-CoV-2 Living Evidence)**  ***Apr 2020***: mental health \| anorexi* \| schizophreni* \| psychos* \| psychot* \| psychiatr* \| depress* \| attempted suicide \| suicid* \| clozapine \| lithium \| bipolar \| anxiety \| National Early Warning Score \| close observation \| seclusion* \| restraint* \| visiting policy \| visiting policies \| trauma \| traumatic \| violence \| aggress* \| agitat* \| eating disorder* \| bulimia \| antipsychotic* \| mood stabili* \| antidepress* \| service* \| personnel \| staff \| self-harm \| self-injury \| NSSI \| DSH \| parasuicide \| passive death wish \| depot \| rapid tranquillisation \| rapid tranquillization \| hypnotic \| GABA-modulator \| antimanic \| ECT \| TMS \| psychotherapy \| group therapy \| CBT \| cognitive behavioural \| cognitive behavioral \| dialectical \| DBT \| mentalisation \| mentalization \| MBT \| sickness absence \| occupational health \| Balint group \| mental health act \| section \| detain \| community treatment order \| section 17 leave \| involuntary institutionalization \| patient safety \| quality of care  **COAP Living evidence on COVID-19***  ***Apr 2021:*** ((mental health) OR (anorexi) OR (schizophreni) OR (psychos) OR (psychot) OR (psychiatr) OR (depress) OR (attempted suicide) OR (suicid) OR (clozapine) OR (lithium) OR (bipolar) OR (anxiety) OR (National Early Warning Score) OR (close observation) OR (seclusion) OR (restraint) OR (visiting policy) OR (visiting policies) OR (trauma) OR (traumatic) OR (violence) OR (aggress) OR (agitat) OR (eating disorder) OR (bulimia) OR (antipsychotic) OR (mood stabili) OR (antidepress) OR (self-harm) OR (self-injury) OR (NSSI) OR (DSH) OR (parasuicide) OR (passive death wish) OR (depot) OR (rapid tranquil) OR (hypnotic) OR (GABA-modulator) OR (antimanic) OR (ECT) OR (TMS) OR (psychotherapy) OR (group therapy) OR (CBT) OR (cognitive behavioural) OR (cognitive behavioral) OR (dialectical) OR (DBT) OR (mentalisation) OR (mentalization) OR (MBT) OR (sickness absence) OR (occupational health) OR (Balint group) OR (mental health act) OR (section) OR (detain) OR (community treatment order) OR (section 17 leave) OR (involuntary institutionalization) OR (patient safety) OR (quality of care)) AND (risk)  ***PubMed, EMBASE, medRxiv and bioXriv electronic bibliographic databases are included in COAP.** |
| --- |
