# Supplementary material for: Impact of the COVID-19 Pandemic on the Global Delivery of Mental Health Services and Telemental Health: Systematic Review
Source: JMIR Ment Health. 2022 Aug 22;9(8):e38600. doi: 10.2196/38600 (PMC9400843; doi:10.2196/38600)

**Multimedia Appendix 2.** PRISMA (Preferred Reporting Items for Systematic Reviews and Meta-Analyses) flowchart.


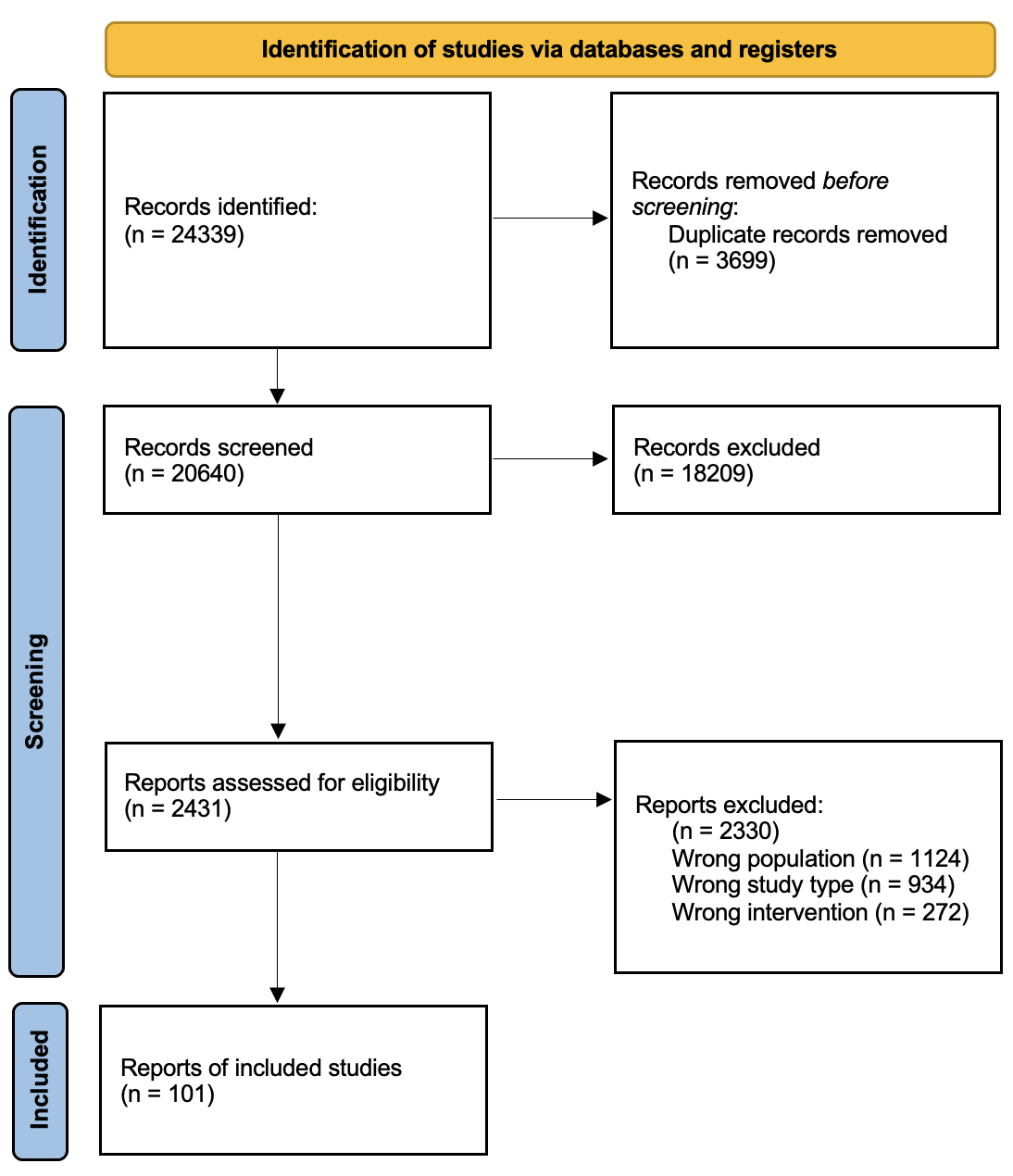

Supplement: Multimedia Appendix 2 [file mental_v9i8e38600_app2.docx]
