# Supplementary material for: Impact of the COVID-19 Pandemic on the Global Delivery of Mental Health Services and Telemental Health: Systematic Review
Source: JMIR Ment Health. 2022 Aug 22;9(8):e38600. doi: 10.2196/38600 (PMC9400843; doi:10.2196/38600)
Supplement: Multimedia Appendix 3 [file mental_v9i8e38600_app3.docx]

**Multimedia Appendix 3.** Impact of COVID-19 on mental health services: summary of key findings.

|  | - ENGLAND: A 26% reduction in inpatient person days was observed for the 31-day periods before and after 16^th^ March in 2020, compared to a 3-6% reduction observed between 2015 and 2019. Virtual contacts increase by 117% compared to a 3-22% reduction observed between 2015 and 2019 [Stewart & Broadbent 2020]. |
| --- | --- |
|  | - ITALY: The number of admissions in 4 Italian departments of mental health decreased by 31.3% in March 2020 to March 2019, and by 25.7% February 2020. The decrease was significant for voluntary admissions (p < 0.001), while the difference was not significant for involuntary admissions (p = 0.87). The number of involuntary admissions, evaluated over the whole considered period, was constant (16 admissions on average per month) [Clerici et al. 2020]. |
|  | - FRANCE: The mean number of consultations in a psychiatric emergency centre in France per day was significantly lower in 2020 (10.8 ± 3.3) than in 2017 (23.3 ± 6.1, p < 10^−15^), 2018 (24.4 ± 5.9, p < 10^−15^), and 2019 (26.4 ± 7.3, p < 10^−15^) [Pham-Scottez et al. 2020]. - FRANCE: The mean number of consultations per day during the confinement was also significantly lower than during the period before the confinement (23.6 ± 6.2, p < 10^−15^) [Pham-Scottez et al. 2020]. |
|  | - UNITED KINGDOM: Comparing data of patients admitted in Leicester acute adult mental health services (UK) during the COVID-19 period (n = 63) and a control period in 2019 (n = 80), more people received a psychotic diagnosis on admission (52% versus 35%) or mania/bipolar affective disorder (25% vs 15%) (x2 = 20.8, p = 0.01) [Abbas et al. 2020]. |
|  | - SPAIN: Comparing 5 weeks before and after the declaration of the state of emergency in Spain, an increased trend in the number of in-person consultations (10 ± 9.08 vs 24.2 ± 6.90, p = 0.024) [Aguilar et al. 2021]. |
|  | - ITALY: A decrease in the utilisation of mental health services specifically dedicated to migrants and individuals with socioeconomic difficulties has been observed in terms of patients accessing the service and follow-up visits after the introduction of lockdown measures compared to control periods from 2017 to 2019 (17.53% vs 30%) [Aragona et al. 2020]. |
|  | - GERMANY & AUSTRIA: During the early phase of the COVID-19 pandemic the number of crisis hotline calls increased both in Austria and in Germany [Arendt et al. 2020]. |
|  | - USA: Comparing incidence of primary emergency department diagnoses for mental, behavioural and neurodevelopmental disorders of the early pandemic period (n = 2913) in Massachusetts (US) with the year before (n = 3892), a reduction of 25.2% was registered (IRR 1.08, 95%CI 1.03-1.13). Similarly, the incidence of subspecialty consultations from the emergency department (n = 2433) was lower than the year before (n = 1925) (-20.9%, IRR 1.15, 95%CI 1.08-1.22) [Baugh et al. 2020]. |
|  | - USA: Psychiatric consultations and patient volume in North Carolina (US) decreased with the onset of the pandemic by 66.9% and 25.2%, respectively. After the introduction of video-consultations, psychiatric consultations were 39.1% lower compared to pre-pandemic data [Beran et al. 2020]. |
|  | - GERMANY: Utilisation of psychiatric inpatient care and subsequent hospitalisation deficit during the COVID-19 pandemic compared to the year before was -18% [-19%, -17%], while a surplus in outpatient care was registered (2% [1%, 2%]). Mean length of stay was slightly shorter (16.8 ± 20.9 vs 17.5 ± 21.5 days; p < 0.01). In-hospital mortality was similar during the COVID-19 pandemic and before (0.4% vs 0.5%, OR 1.01, 95%CI 0.78, 1.31; p < 0.95) [Bollmann et al. 2021]. |
|  | - UNITED KINGDOM: Comparing the service use in a London hospital during the early phase of the COVID-19 pandemic and the year before, a 40% reduction in the number of psychiatric referrals (404 vs 241) were registered [Butler et al. 2021]. |
|  | - ITALY: During the early COVID-19 pandemic, the voluntary admissions rate (3.28 per 100000 habitants) was significantly reduced compared to the same period in 2019 (5.98 per 100000 habitants; p < 0.001) in Friuli Venezia Giulia (Italy). Non-significant changes were recorded for voluntary admissions to psychiatric outpatient services between the 2019 (12.36 per 100000) and 2020 (8.98 per 100000; p = 0.41) [Castelpietra et al. 2021]. |
|  | - UNITED KINGDOM: During the early COVID-19 pandemic in Cambridgeshire and Peterborough (UK), there was a sharp reduction at lockdown in referrals to primary care MH services (p < 0.001), psychological therapy (p < 0.001), and all secondary care MH teams (p < 0.001) apart from EIP services (p = ns), with a subsequent increase towards normal levels for secondary care services (p < 0.01-0.001) [Chen et al. 2020b]. |
|  | - USA: Daily tele-mental health visits rose from 1739 to 11406 (+556%) in the weeks following the pandemic declaration within the Department of Veterans Affairs (USA). Daily in-person encounters fell from 57296 to 10931 (-81%) [Connoly et al. 2020]. |
|  | - USA: In an American eating disorder multidisciplinary unit, the yearly average inpatient census by month changed from 31.92 (SD = 5.33; before the COVID-19 pandemic) to 19.33 (SD = 4.5; March 2020 to May 2020) (59% of the usual census) with a shift to a full telehealth system. [Datta et al. 2020]. |
|  | - ITALY: Comparing data from early COVID-19 pandemic and the year before in Modena (Italy), urgent psychiatric consultations increased from 656 to 811 (+24%) [Di Lorenzo et al. 2021]. |
|  | - SPAIN: Comparing data of an adolescent acute psychiatric unit in Madrid (Spain), 25 children or adolescents required an evaluation at the psychiatric emergence service in 2020 compared to 64 in 2019 (-61%), while 18 adolescents in 2020 were hospitalized against 31 in 2019 (-42%) [Diaz de Neira 2021]. |
|  | - UNITED KINGDOM: Comparing the number of referrals during March-May 2020 at the Child and Adolescent Mental Health Urgent Consult Clinic (UK) with March-May 2019, the number of referrals decreased from 84 to 63 [Dolp et al. 2020]. |
|  | - USA: During the early COVID-19 pandemic in New York (USA), the number of emergency psychiatric evaluation decreased by 43% (n = 201, p < 0.001) compared to data from the previous months (n = 355). Both children/adolescents (31.7% vs 50.8%, p = 0.02) and adults (52.9% vs 66.2%, p = 0.04) were more likely to be admitted as inpatients compared to the control period [Ferrando et al. 2020]. |
|  | - USA: Comparing children and adolescents’ access to a psychogenic nonepileptic events clinic in Ohio (USA) during the first 6 months of 2020 to the same period of 2019, the number of visits slightly decreased (40 vs 45) [Fredwall et al. 2021]. |
|  | - CHILE: During the early COVID-19 pandemic in a large private academic health network in Santiago (Chile), 24.7% of the visits delivered in 2019 have been delivered via telepsychiatry [Garcia-Huidobro et al. 2020]. |
|  | - INDIA: In India a telemedicine-assisted stepped-care outpatient service for substance use disorder was developed and started on 18^th^ of May 2020. Till 31^st^ August 2020, 160 new and 219 follow-up patients were registered in the tele-addiction service. Among them, 128 (80%) and 198 (90.2%) patients received teleconsultations, respectively. A total of 28 received a physical consultation. [Ghosh et al. 2021]. - INDIA: Compared to 2019, the number of patients with OPD dropped (May 170 vs 22; June 351 vs 43; July 467 vs 44; August 436 vs 51). [Ghosh et al. 2021] |
|  | - USA: The number of emergency department visits at the large hospital in Utah (USA) during the early COVID-19 pandemic and the previous year were compared for different clinical conditions: suicide and self-inflicted injuries, +0.5% (820 vs 738); mood and personality disorders, -0.4% (280 vs 431) [Giannouchos et al. 2020]. |
|  | - SPAIN: Comparing the early lockdown period to the previous 3 months, the number of psychiatric emergency admissions in Barcelona (Spain) decreased from 1208 to 750 (-37.9%, 95%CI -37.7, -55.8, p < 0.01) [Gomez-Ramiro et al. 2021]. |
|  | - USA: Comparing the three months after COVID-19 was declared a national emergency in USA (March-May 2020) to the year before, the monthly average number of emergency department visits decreased from 5126.9 to 3306.7 between the pre-pandemic and pandemic periods (−1820.3; 95%CI −3406.3, −234.2). The monthly average percentage of patients screening positive for opioid use disorder were not significantly different (4.3 vs 4.6; +0.3; 95%CI −0.4, 1.0), as the average number of monthly ED visits for overdose (17.7 vs 20.3; +2.6; 95%CI −4.8, 10.1) and the average monthly proportion of ED visits for overdose (0.4% vs 0.7%; +0.3; 95%CI −0.3, 0.9). Monthly state overdose fatalities increased from 9.4 to 15.3 (+5.9; 95%CI 0.8, 11.1) [Grunvald et al. 2021]. |
|  | - SWEDEN: Comparing data on the patients accessing a regional Gambling Disorder Unit in Malmö (Sweden) during and before the COVID-19 pandemic, the number of unique patients seen (p = 0.72), the number of unique female (p = 0.43) and male (p = 0.97) patients, the total number of contacts (p = 0.65), and the number of no-shows (p = 0.49) were not significantly affected by the COVID-19 pandemic. The number of changes/cancellations decreased (p < 0.01) compared to the period before the COVID-19 pandemic [Hakansson et al. 2021]. |
|  | - USA: Data from 52 hospitals showed a percent decrease from the expected to the observed number of patients was estimated: 37% (from 1053 to 667) decrease in the number of patients identified with opioid use disorder, 34% (from 632 to 420) decrease in the number of patients who accepted a referral, 48% (from 521 to 272) decrease in the number of patients who were prescribed buprenorphine, 53% (from 501 to 234) decrease in the number of patients who were administered buprenorphine, and 33% (from 416 to 277) decrease in the number of patients who attended at least one follow-up visit for addiction treatment [Herring et al. 2021]. |
|  | - USA: Comparing delivery of tele-mental health services in New York (USA) before, during and after the lockdown, adults (n = 1115, 21131 sessions) showed a preference towards telehealth compared to child (n = 1374, 22163 sessions) (p < 0.001), with a relative decrease in the number of child mental health services with the switch to telehealth in March 2020 (onset of lockdown) and a relatively rapid shift back to face-to-face among child services when in-person services resumed in May and June 2020 (χ2 = 21.745, p < 0.01) [Hoffnung et al. 2021]. |
|  | - AUSTRALIA: Comparing patients accessing inpatient psychiatric treatment in two councils of Melbourne (Australia) during the COVID-19 pandemic (March-May 2020) and before (March-May 2019), the number of admissions were comparable (104 and 109, respectively) [Itrat et al. 2021]. |
|  | - AUSTRALIA: Comparing patients accessing inpatient psychiatric treatment in the north and west of the metropolitan Melbourne (Australia) during the COVID-19 pandemic (March-September 2020) and before (March-September 2019), the number of admissions were comparable (1817 and 1843, respectively) [Jagadheesan et al. 2021]. |
|  | - USA: The number of visits related to opioid use disorder at an emergency department in Los Angeles (USA) increased from 59 and 35 per 10000 in April and May 2020 compared to 24 and 32 per 10000 in January and February 2020 [Johnson et al. 2020b]. |
|  | - FINLAND: Comparing data from the three largest Finnish Hospitals, the number of ED presentations due to a psychiatric disorder reduced of 18.6% between the 6 weeks before (n= 1024) and after (n= 834) the start of national emergency the 16th of March 2020 [Kuitunen et al. 2020]. |
|  | - HONG KONG: The admission rate of the five psychogeriatric units in Hong Kong increased of 21.4% (IRR 1.21 95%CI 0.96-1.54, p = 0.11) in the first pandemic period (February – April 2020) compared to the pre-pandemic one (November 2019 – January 2020) - 153 and 126 admissions, respectively [Lee et al. 2020]. |
|  | - CHINA: During the pandemic the Guangdong Second Provincial General Hospital and Internet Hospital (China) integrated a web-based COVID-19 platform which included psychological support. Between 23rd of January and 31st of March 2020, the psychological counselling program served 474 users (7 uses per day) while in the post-epidemic period (1st April – 30th June 2020) the number reduced to 162 (2 uses per day) [Lian et al. 2020]. |
|  | - AUSTRALIA: Comparing data of private psychiatrists’ visits held in April-June 2020 and the same period of 2019 in Australia, the number of psychiatry consultations (telehealth and face-to-face) rose during the pandemic by 14%, with telehealth nearly a half of this total. Face-to-face consultations in 2020 were only 56% of the comparative number of 2019 consultations. Most telehealth involved short telephone consultations of ⩽ 15–30 min. Video consultations comprised 38% of total telehealth provision [Looi et al. 2020a]. |
|  | - AUSTRALIA: Comparing data of visits held in Australian rural settings (Australian Capital Territory - ACT, Northern Territory - NT, South Australia – SA, and Tasmania - TAS), the overall rate of consultations (face-to-face and telehealth) increased during March and April 2020, compared to the monthly face-to-face consultation average of July 2018 – June 2019, excepting Tasmania (ACT: 1724 in April, increased 114%; 2061 in May, increased 136% - NT: 296 in April, increased 108%; 337 in May, increased 123% - SA: 12864 in April, increased 116%; 12876 in May, increased 116% - TAS: 1886 in April, reduced 85%; 2189 in May, reduced 98%). For total video and telephone telehealth consultations combined, video consultations were lower in April and higher in May [Looi et al. 2020b]. |
|  | - AUSTRALIA: The total combined use of telehealth and face-to-face private psychiatric consultation services in New South Wales, Queensland, Victoria, and Western Australia regions (Australia) in April and May 2020 increased by 10%−20% of the average monthly face-to-face consultations in the 2018/19 financial year [Looi et al. 2020d]. |
|  | - AUSTRALIA: Comparing data of private psychiatrists’ visits held in July-September 2020 and the same period of 2019 in Australia, the number of psychiatry consultations (telehealth and face-to-face) rose during the pandemic by 14%, with telehealth 43% of this total. Face-to-face consultations in 2020 were only 64% of the comparative number of 2019 consultations. Most telehealth involved short telephone consultations of ⩽ 15–30 min. Video consultations comprised 42% of total telehealth provision [Looi et al. 2021]. |
|  | - QATAR: Analysing data on the number of visits in the mental health outpatient setting in Qatar in the four months following the introduction of telepsychiatry (March-June 2020), the total number of individuals accessing mental health services increased by approximately 36.5% compared to the same period in 2019 (22086 and 16175 cases, respectively) [Karim et al. 2020]. |
|  | - JAPAN: When Japan declared the state of emergency (April 2020), national collected data on the average length of hospital stays registered an exceeding above the 95% upper value with an excess between 3 and 35 days (0.85% -13.45%). An excess has been registered also in May with excess values = 28 - 60 days (10.71 % - 23.24%. In the same month, values below the 95% lower bound were observed in the number of daily outpatient visits (average 6898 –11014, 11.67% – 18.64%) [Makiyama et al. 2021]. |
|  | - USA: Data from the Johnson Depression Center and the Steven A. Cohen Military Family Clinic, at the University of Colorado Anschutz Medical Campus (US), showed that the change to tele-mental health helped in reduced the no-show rates from 11.4% (n= 57) in the 2 weeks before the pandemic (2nd-13th March 2020) to 7.8% (n= 38) after the virtualisation (30th March – 10th April). In the following months (April – September 2020) the no-show rate has been stable with a rate between 5.5% and 8.5%, and an 26.2% increase in the overall completed visits [Mishkind et al. 2020]. |
|  | - USA: The Medical University of South Carolina’s Women’s Reproductive Behavioral Health Program provides outpatient mental health and substance use treatment to pregnant and postpartum women within obstetric practices. After the onset of the COVID-19 (April-May 2020), the average monthly utilisation of telehealth service increased by 90% [Moreland et al. 2021]. |
|  | - UNITED KINGDOM: Comparing data in Northamptonshire, UK, before (November 2019) and during the pandemic (April 2020), the ED psychiatric attendance fell by 49% (from 19443 to 9904 visits) whereas referrals to liaison psychiatry remained unchanged (November 2019: 235 referrals; mean daily 7.8, SD = 3; April 2020: 250 referrals; mean daily 8.3, SD = 3) [Mustafa et al. 2020]. |
|  | - USA: Data compared the number of MH consultations visit made to cancer patients held face-to-face in April-September 2019 and both face-to-face and virtually in April-September 2020. In 2020 the number on inpatient consultations (330 vs 623), referrals (127 vs 175), and outpatient visits (448 vs 550) decreased. Within the outpatient visit, 359 were telephone contacts, 69 video visits, and only 20 in person [Rainwater et al. 2021]. |
|  | - SPAIN: Comparing variations in activity of 2020 and 2019 in Salamanca (Spain), a reduction was observed across several units: short-term hospitalisation unit (-51%), day hospital (-67%), liaison psychiatry (-14%), psychiatric emergencies room (-64%), addictive disorder unit (-87%), child and adolescence mental health team (-99.7%), community mental health team (-90.6%), and severe mental illness program (-93.75%). The only unit with a recorded increase in activity from 2019 was convalescence unit (+10%) [Roncero et al. 2020a]. |
|  | - USA: This study reported data from the Veterans Health Administration (VHA), the largest integrated health care system in the United States. Before the pandemic (Oct 2019 0 Feb 2020) VHA delivered from 1.5 to 1.8 million visit per month, with 85% of in person visits, 11% by telephone, and 5% by video. The use of telepsychiatry increased with the pandemic and in June 2020 they provided 1.5 million visits of which 19% in person, 59% by telephone, and 20% by video [Rosen et al. 2020]. |
|  | - IRAN: The number of psychiatric interventions in an emergency medical service in Teheran (Iran) reduced of 27% (p<0.001), comparing the period before (21st Jan – 17th Feb 2020, n= 5545) and after the COVID-19 outbreak (18th Feb – 16th Mar 2020, n= 4057) [Saberian et al. 2020]. |
|  | - ISRAEL: During the 2020, the total number of psychiatric consultations provided by the staff of the division of psychiatry of Tel Aviv Sourasky Medical Center (Israel) to the Emergency department dropped to an average of 262 per month compared to the previous 4 years (2016-2019), in which the average number of consultations ranged between 381 (2016) and 339 (2019) [Schreiber et al. 2021]. |
|  | - ITALY: Data from a memory clinic in Rome (Italy) shows that 72% of appointments (n= 251) were cancelled during the lockdown period (i.e., March-April 2020). Comparing this with 2019 figures, there was a significant difference in the proportion of cancelled follow-up (p<0.001) in both March (70.4% vs 17.3%) and April (84.4% vs 27.3%), and first-time (p=0.001) appointments in March (76.2% vs 36.4%) [Spalletta et al. 2020]. |
|  | - AUSTRALIA: In Australia, the use of the MindSpot Clinic, a national digital mental health service (DMHS) providing services to people experiencing anxiety and depression, during the pandemic (19th March - 10th June 2020), increased of 16.7% (mean number assessment per week = 455) compared to the period between 1st and 28th September 2019 (390 assessments per week) [Staples et al. 2020]. |
|  | - USA: Data collected from an inpatient unit of an Academic Medical Center in New Haven (US) shows that from March to July 2020 they admitted 964 patients, with a decreased of 30-40% in the inpatient rate during the peak COVID weeks [Li et al. 2021b]. |
|  | - UNITED KINGDOM: A total of 48739 patients had an index episode of self-harm recorded in England during the period between 10th March and 10th June of the previous 10 years (2010-2019), while 4238 were recorded in the COVID-19 first-wave cohort (10th March – 10th June 2020). The likelihood of receiving a remote GP/practice nurse consultation within 3 months of a self-harm episode was higher in the COVID-19 pandemic (67.7%) than in the pre-pandemic period (32.3%, ratio 2.10, CI 2.05-2.15), but the overall likelihood of having a GP/practice nurse consultation was slightly lower (80.3% vs 83.2%, ratio 0.97, CI 0.96-0.98) [Steeg et al. 2021]. |
|  | - AUSTRALIA: The Mental Health service volume activity reduced of 3.3% in the period March-June 2020 compared to the same period of 2019 in New South Wales (Australia). The Emergency department presentation decreased in the first 3 months (-8%, -13%, and -1%, respectively) and increased in June (+6%) compared to the previous year [Sutherland et al. 2020]. |
|  | - AUSTRALIA: Evaluating data from two Australian Emergency department services, consisting of 3299 presentations made by 2153 persons before COVID-19 (March–August 2019), and 3190 presentations made by 2287 persons since COVID-19 (March–August 2020), the authors reported an increment in discharges following suicidal and self-harm presentations (78.9% vs 74.4%, p<.001), and a reduction in the hospital admissions (17.9% vs 19.9%, p=.038) [Sveticic et al. 2021]. |
|  | - USA: During the COVID-19 period (March 11 to August 31, 2020), an inpatient child and adolescent unit in Texas registered a 41% reduction in the number of self-referral presentations and a 36.5% reduction in the number of patients transferred from other hospitals, which accounted for an overall 40% reduction in admissions during the COVID-19 period [Ugueto et al. 2021]. |
|  | - ISRAEL: Data about the treatment of adolescents with eating disorder (ED) in a paediatric ED-treatment centre in Israel shows that the number of patients treated (242 vs 257) and of new cases (127 vs 166) during the COVID-19 outbreak in the first 10 months of 2020 was slightly lower than the respective period in the past five years (2015– 2019). Patients and families received more multidisciplinary sessions (5926 sessions), compared to the pre-pandemic period (4001 sessions). Telemedicine meetings comprised of 37% of all sessions during 2020 (2193 sessions), vs no use during the respective period between 2015 and 2019 [Yaffa et al. 2021]. |
|  | - USA: The number of patients seen for a MH/SUD visit in Massachusetts, in the January–June period 2020, was slightly increase (n= 73184) compared to the same period of 2019 (n= 62756) and 2018 (n= 52907). During the surge (March 25th - May 18th, 2020) of the pandemic, MH/SUD visits increased of 9.1% compared to pre-COVID-19 levels (p < 0.05), while during the partial reopening period (May 19th - June 30th, 2020) they decreased, although not statistically, to below pre-pandemic 2020 levels (−12.1%) [Yang et al. 2020]. |
|  | - GERMANY: Emergency service presentation rates decreased significantly by 26.6% (RR 0.734 95%CI 0.58, 0.93, p = 0.009) between early (weeks 1-11) and late (weeks 12-15) epochs in 2019 and 2020 in a large psychiatric hospital in Germany, reflecting the impact of the COVID-19 pandemic [Hoyer et al. 2020]. |
|  | - UNITED KINGDOM: Working daily contacts with community mental health teams in London were 702 ± 59 prior to 16th March and 599 ± 139 (-14.6%); specifically, these were 548 ± 62 versus 219 ± 70 respectively for face-to-face contacts (-60.0%), and 154 ± 17 versus 380 ± 97 for virtual contacts (+147%). Daily caseloads reduced by 2.1% 8,729 ± 24 versus 8,539 ± 124) [Stewart et al. 2020]. - UNITED KINGDOM: Comparing the period before 16th March to that between 16th March and 15th May for home treatment teams, face-to-face contacts decreased by 50.2% (135.2 ± 26.8 versus 67.4 ± 18.0), mean virtual contacts increased by 102.7% (26.7 ± 7.3 versus 54.2 ± 14.8), mean total contacts reduced by 24.9% (161.9 ± 30.7 versus 121.5 ± 26.1), and mean daily caseload decreased by 26.4% (221.8 ± 8.5 versus 163.3 ± 20.0). Mean daily caseloads increased by 12.2% (158.6 ± 19.2 versus 177.9 ± 15.1) [Stewart et al. 2020]. |
|  | - BRAZIL: Salum and colleagues reported the impact of the COVID-19 pandemic on a mental health community centre in Brazil with 154 patients. Phone contact was possible for 61% of patients, with 29% being advised face-to-face through regular service attendance, and around 7% of service users could not be contacted. Overall, intensive case management via telehealth is a feasible strategy that can be used in low- and middle-income countries [Salum et al. 2020]. |
|  | - USA: Sharma and colleagues registered an overall reduced number of study visits (645 versus 358) over a 1-month period with a shift towards phone and home-based tele-mental health (n = 171 and n = 160, respectively) compared to in person visits (n =27) [Sharma et al. 2020]. |
|  | - GREECE: In a report from a mental health helpline service in Greece, most calls pertained to the quarantine (n = 482, 83.7%), and more specifically to feelings of restraint (56%) and loneliness (52.9%) [Peppou et al. 2020]. |
|  | - ITALY: Carpiniello and colleagues presented data from 71 departments of mental health and 107 psychiatric wards in general hospitals on the early impact of COVID-19 pandemic in Italy. 14% of community mental health centres closed and approximately 25% reduced their hours of access. A decrease was registered (approximately -78%) in the number of operational day hospitals, which are largely involved in the clinical monitoring and treatment of subacute but not severe cases, whilst an even greater reduction (-85%) has been observed in the number of operational day centres focusing on psychosocial and rehabilitation activities [Carpiniello et al. 2020a]. - ITALY: Scheduled psychiatric consultations, both at home and on-site, went ahead for selected cases, being replaced in approximately 75% of cases by scheduled remote contact, mainly phone calls (100%), videocalls (67%) or emails (19%), with 41% of units adopting all these means of contact [Carpiniello et al. 2020a]. - ITALY: All other activities were affected by a significant decrease, including psychiatric consultations for general hospitals (approximately -25%), individual psychotherapies (approximately -60%), group psychotherapies and psychosocial interventions (approximately -90% and -95%), and monitoring of cases manifested in residential facilities (-40%) and among offenders affected by mental disorders assigned by the Court to community mental health centres (-40%) [Carpiniello et al. 2020a]. |
|  | - ITALY: In Italy during the early COVID-19 pandemic, 15.5% of the psychiatric outpatient services (n = 71) were closed and 26.8% had introduced restricted access hours. Scheduled (on-site 88.7%, at home 83.0%) and urgent (on-site 100%, at home 95.7%) psychiatric visits were delivered, while a substantial impact was recorded for individual (46.4%) and group (5.6%) psychotherapies and psychosocial interventions (1.4%). 13.1% of the surveyed general hospital psychiatric ward (n = 107) were closed, recorded a reduced number of beds (31.8%) or admissions (87.8%) [Carpiniello et al. 2020b]. |
|  | - CHINA: A Chinese survey on 33 hospitals providing child and adolescents mental health inpatient and outpatient services was conducted in early March 2020 (out of a total of 50 similar services available nationwide). According to this national survey, 9% (n = 3) of hospitals closed their outpatient units [Cui et al. 2020]. - CHINA: The total number of outpatient visits dropped to 53% compared to before the COVID-19 pandemic [Cui et al. 2020]. - CHINA: Inpatient referrals declined by 50% and nearly 25% of hospitals closed inpatient wards [Cui et al. 2020]. - CHINA: A correlation analysis on the impact of the distance from Wuhan on percent reduction of visits found a significant inverse correlation for outpatients (r = -0.463, p < 0.01) and even more so for inpatients (r = -0.709, p < 0.001) [Cui et al. 2020]. |
|  | - AUSTRIA: An online survey on 1,547 psychotherapists in Austria found that the overall number of patients treated on average per week in the early weeks of the COVID-19 lockdown (10.1 ± 9.1) was significantly lower compared to the months before the COVID-19 lockdown (14.0 ± 11.3, -28%, p < 0.001), as the number of patients treat in personal contact (13.5 ± 10.6 versus 2.6 ± 4.8, -81%, p < 0.001) [Probst et al. 2020]. - AUSTRIA: The number of patients treated on average per week via telephone overall increased (0.42 ± 3.01 versus 4.53 ± 5.77, + 979%, p < 0.001), as those treated via Internet (0.18 ± 1.35 versus 2.99 ± 4.44, + 1561%, p < 0.001) [Probst et al. 2020]. |
|  | - USA: An online survey involving 93 clinic directors or designated representatives of Association of Psychology Training Clinics mainly in the USA (n = 89) showed that 23.7% (n = 22) was forced to close and discontinue services, at least temporarily. Of those remained open for services, 61 training clinics (65.6% of the total sample, 86% of those remained open) reported that their sites remained open primarily using telepsychology [Hames et al. 2020]. |
|  | - CZECH REPUBLIC, GERMANY & SLOVAKIA: A survey on 338 healthcare professionals in Czech Republic, Germany and Slovakia showed that the number of patients treated on average per week in personal contact decreased from 17.84 ± 12.02 to 9.20 ± 10.35 per week (-48%, t 13.224, p < 0.001) [Humer et al. 2020a]. - CZECH REPUBLIC, GERMANY & SLOVAKIA: On the other hand, the number of patients treated via telephone increased from 0.92 ± 3.16 to 3.28 ± 5.22 per week (+257%, t -8.717, p < 0.001), and the number of patients treated via internet increased from 0.59 ± 2.54 to 5.83 ± 6.82 per week (+888%, t -15.346, p < 0.001) [Humer et al. 2020a]. |
|  | - SPAIN: 9,038 phone interviews were carried out in the outpatients and community mental health programs to describe the response of the Mental Health Network of the Salamanca Area (Spain) to the COVID-19 pandemic. The activity in subacute and acute wards, as well as that of the day hospital programs decreased by 50% [Roncero et al. 2020b]. |
|  | - FRANCE: In France, 91% of the 65 ECT centres participating an online survey experienced a decrease in their activity. 47% centres experienced a total cessation of activity and 27% of the centres experienced a decrease of more than half of their usual activity [Amad et al. 2020]. |
|  | - UNITED KINGDOM: 61.1% of 2,180 mental healthcare workers in the UK rated the adoption of new digital ways of working as very or extremely important in the management of COVID-19 impact at work [Johnson et al. 2020a]. - UNITED KINGDOM: A survey on 2,180 mental healthcare workers in the UK provided insight on the challenges at work during the COVID-19 pandemic: the risk of spreading COVID-19 between service users and colleagues in inpatient facilities (65.3% and 60.4% rated very or extremely relevant out of 643 and 641 respondents), lack of activities and facilities leading to increased boredom and agitation in inpatient facilities (63.4% rated very or extremely relevant out of 533 respondents), challenging environment because of social restrictions in residential services (76.2% rated very or extremely relevant out of 63 respondents), and having to adapt too quickly to new ways of working for community teams and psychological treatment services (55.0% rated very or extremely relevant out of 1261 respondents) [Johnson et al. 2020a]. |
|  | - AUSTRALIA: Zulfic and colleagues conducted an audit of 314 community patients to examine the potential implications of a move to predominantly telephone support. In all, 21 (7%) did not have access to a phone, and a further 58 (18%) were deemed unreliable in responding to contact over the phone based on past clinician experience. The majority of these difficult-to-reach patients had a diagnosis of schizophrenia or schizoaffective disorder (83%), and 75% were considered high risk for COVID-19 complications [Zulfic et al. 2020]. - AUSTRALIA: There was a group of patients that require regular face-to-face reviews, including the 91 patients (29%) who are treated with depot medications and 71 (23%) taking clozapine [Zulfic et al. 2020]. |
|  | - FRANCE: In France, a health care and education centre for adolescents which provides long-term psychiatric care, created a virtual ward using Discord to ensure continuity of care. Of the 38 patients benefitting from this service, 1 stopped early and 5 withdrew” [Berthaut & Chamignon 2021]. |
|  | - ITALY: 42.1% (n = 130) of the psychotherapists surveyed in Italy (n = 308) reported that their psychotherapy treatments were interrupted during the COVID-19 lockdown. Only 7.2% of their treatments (n = 22) were delivered face-to-face, while taking precautionary measures (e.g., wearing masks and gloves) [Boldrini et al. 2020]. |
|  | - ITALY: A Psycho-oncology unit in Italy reported to have treated 28 cancer patients, 9 caregivers, and 7 family members during the lockdown period (Apr-Sep 2020) switching from face-to-face visits to virtual consultations [Cantini et al. 2020]. |
|  | - USA: In the USA 39-year-old female individual with non-responsive schizoaffective disorder with catatonia, dependent personality disorder and substance use disorder had her treatment with ECT interrupted after the second session because of the COVID-19 pandemic [Casano et al. 2020]. |
|  | - ITALY: In Emilia Romagna region (Italy), a free phone-based psychological support was established, receiving 312 phone calls in the first 11 weeks [Ghiretti et al. 2020]. |
|  | - SPAIN: During the early COVID-19 pandemic in a hospital in Madrid (Spain), out of 1818 outpatient consultations carried out, 1329 (73.10%) were delivered by telephone or videoconferencing and 489 (26.9%) face-to-face, corresponding to 365 patients who were receiving treatment at the time in the outpatient clinic or day hospital [Graell et al. 2020]. |
|  | - INDIA: A survey compared the services in both public and private institutes during the lockdown period in India (n = 109). All the services other than telecommunication services (19.3% vs 45.9%) decreased: outpatient services (100.0% vs 67.9%), inpatient services (94.5% vs 57.8%), ECT services (75.2% vs 21.1%), brain stimulation services (14.7% vs 0.9%), psychiatry emergency services (91.7% vs 82.6%), psychiatry consultation-liaison services (93.6% vs 75.6%), opioid substitution therapy services (31.2% vs 22.0%), psychotherapy services (79.8% vs 33.0%), and psychological investigations (75.2% vs 25.7%) [Grover et al. 2020b]. |
|  | - INDIA: A survey compared the services in private sector provided during the lockdown period in India (n = 396). All the services other than telecommunication services (26.3% vs 52.0%) decreased: outpatient services (100.0% vs 58.8%), inpatient services (38.6% vs 16.2%), ECT services (30.8% vs 7.8%), brain stimulation services (9.1% vs 3.0%), psychiatry emergency services (47.7% vs 37.6%), psychiatry consultation-liaison services (64.6% vs 27.8%), opioid substitution therapy services (17.2% vs 6.8%), psychotherapy services (61.6% vs 16.7%), and psychological investigations (41.4% vs 6.8%) [Grover et al. 2020c]. |
|  | - USA: Of the 818 mental health care professionals in the US who reported using telepsychiatry during the pandemic (April-May 2020), 500 (61%) used both video and telephone, 273 (33%) only telephone, and 45 (6%) only video. Amongst the advantages, flexible scheduling or rescheduling, timely appointment starts, and lack or reduction of no-shows were reported [Guinart et al. 2021]. |
|  | - USA: In New York (USA), a telephonic-based model was implemented to continue to address substance use within emergency departments. In 13 weeks, there were 228 incoming calls, 190 outgoing calls, and 4 voicemails. 108 (26%) of calls were with patients, 13 (3%) with family/friends, 224 (53%) with staff members, and 79 (19%) with treatment providers [Harrison et al. 2020]. |
|  | - INDIA: In the Assam region (India), a psychological helpline received 239 calls over the 18 days of lockdown (7-24 April 2020) [Hazarika et al. 2021]. |
|  | - INDIA: In a large psychiatric hospital in India telepsychiatry service has been offered after the lockdown in March 2020. Between April and September, they did 168 consultations. Number of teleconsultations during lockdown was expectedly positively correlated with travel cost saved (ρ = 0.47, p <0.01) and time saved (ρ =0.49, p <0.01) [Khanra et al. 2021]. |
|  | - CANADA: A Canadian survey, directed to Direct Support Professionals (n= 942) working during the pandemic with people with intellectual and development disabilities (IDD), reported that the majority completed telephone-based visits (58%, n= 549), 24% of professionals attended a face-to-face visit (n= 225), while only 22% made at least one videocall (n= 204) [Lunsky et al. 2021]. |
|  | - SPAIN: During the lockdown in Spain, a team of psychiatrist in Salamanca visited a shelter for homeless people to provide psychiatric consultations. Between the 27th of March and the 25th of May 2020, they attended the shelter 7 times providing 50 psychiatric visits. They also contacted the shelter by phone (7 calls) or email (3 contacts) to provide further follow up [Martin et al. 2020]. |
|  | - USA: The “Addiction Telehealth Program” (ATP) is a telephone-based program to reduce treatment access barriers for people with substance use disorders staying at San Francisco’s COVID-19 Isolation and Quarantine (I&Q) site. In the 2 weeks before the implementation of ATP for I&Q guests, the program received 10 calls from other health care providers. In the 6 weeks after the implementation (10th April – 25th May) they provided 59 consultations for the I&Q sites [Mehtani et al. 2021]. |
|  | - USA: During the pandemic (March-September 2020) a psychosocial oncology service in the US continued to provide visits through telepsychology for 93% of their established patients (n= 85), while 4 patients left the service and 2 patients preferred to wait for a face-to-face visit. They also received 263 new referrals and delivered a first visit for the 50.6% (n = 133), with 82% (n= 109) of digital visit and 18% (n= 24) face-to-face [Myers Virtue et al. 2021]. |
|  | - INDIA: An outpatient service in India switched their service to telepsychiatry during the lockdown period (25th March – 31st May 2020), delivering 60.1% of the planned follow-up visits (1049 of 1748) [Naik et al. 2021]. |
|  | - DOMINICAN REPUBLIC: In the Dominican Republic a team of volunteers consisting in 598 psychologists and 70 psychiatrists provide telephone counselling during the pandemic. In the period from 25th of March and 17th of May they did 6800 phone interventions [Peralta et al. 2020]. |
|  | - ITALY: In Italy a telephone psychological counselling service was established during the COVID-19 pandemic, totalling 193 users between April and June 2020 [Perricone et al. 2021]. |
|  | - USA: A survey directed to US psychologists showed that the use of telepsychology increased with the pandemic, compared to prior to the pandemic (before January 2020), from 7.07% (sd 14.86) to 85.53% (sd 29.24) of the total activity [Pierce et al. 2020]. |
|  | - ITALY: In Treviso (Italy), the oncology unit switched their psychological monitoring to a virtual modality during the pandemic. In the first 9 weeks, they did 123 on-line visits (12% were videocalls using Google Hangouts, and 88% telephone calls) [Prior et al. 2020]. |
|  | - INDIA: During the pandemic the Indian Government established a helpline for COVID-19 related mental health issues. During the first month of activities (April 2020) 20475 calls were answered by Frontline MH professionals [Ravindran et al. 2020]. |
|  | - USA: In a survey about the use of telepsychology in the US, data shows that most therapists who already used telepsychology before COVID-19 reported an overall increase in requests for therapy services from current patients (36.5%), whereas 41% of those that started to use telepsychology during the pandemic report an overall decrease. As for new clients in both groups the main part reported fewer requests (45% and 53%, respectively) [Sampaio et al. 2021]. |
|  | - INDIA: In India, the Department of Psychiatry of the Sikkim Manipal Institute of Medical Sciences started a new Telepsychiatry services from 25th of March 2020. On the 26th of May 2020, they already had 78 visits with 6 acute cases that needed a hospital referral [Singh Bhandari et al. 2020]. |
|  | - CANADA: During the pandemic in Ontario (Canada), the implementation of a virtual emergency psychiatric consultation service (i.e., Emergency Diversion Clinic) allowed to provide consultations for 60% of all youth presenting to the Emergency Department for a mental health issue. After the assessment, 56% of patients were linked to community mental health support [Hall & Sukhera 2020]. |
|  | - USA: During the early phase of the pandemic, the Homeless Health Care Los Angeles (California, US) implemented a novel “telephone booth” model that allowed socially distanced on-site “face-to-face” treatment of syringe exchange patient. Specifically, before this implementation (from January to 12th March 2020), the twice-weekly medication for addiction treatment (MAT) program served approximately 5–8 patients per week. After the implementation, the patient demand reduced to 1 patient per week at first, with a surged back to pre-pandemic level in May. In the same period, the most common buprenorphine dispensing method changed from a direct dispensing (86% of patients in February 2020) to a through-pharmacy method (89% in April-May 2020) [Tringale & Subica 2020]. |
|  | - TURKEY: Out of a total of 892 patients admitted for COVID-19 symptoms in a hospital of Istanbul (Turkey) between the 10th of March and the 26th of June 2020, 89 patients requested psychiatric consultations [Turan et al. 2021]. |
|  | - GERMANY: In South Germany, a telephone hotline for psychological first aid for COVID-19-related burdens was set from April to July 2020. Of the 8096 calls made, a total of 1292 included a telephone consultation (16%). In 42% of all consultations, the counsellors stated that they had carried out short therapeutic interventions (e.g., supportive conversation techniques, instructions on relaxation, emotion-focused interventions, cognitive interventions, behavioural interventions, psycho-educational interventions, support in problem solving, and resource activation). In 26% of all documented consultations, psychotherapeutic treatment was recommended, while in 11% reference to other specialized telephone services was made [Vonderlin et al. 2021]. |
|  | - CHINA: During the early pandemic phase, in China 1.9% of survey respondents sought help from a mental health specialist (n= 108). Of them, 42.6% attend an Internet-based service (n= 46), 37.0% a face-to-face visit (n= 40), and 20.4% a telephone-based service (n= 22) [Zhong et al. 2020]. |

Categorisation of evidence:

|  | *randomised trial* |
| --- | --- |
|  | *cohort study* |
|  | *before-after study* |
|  | *cross-sectional study* |
|  | *case control study* |
|  | *survey/audit, case series, case report* |

References

1. Abbas, M. J., Kronenberg, G., McBride, M., Chari, D., Alam, F., Mukaetova-Ladinska, E., Al-Uzri, M., & Brugha, T. (2021). The Early Impact of the COVID-19 Pandemic on Acute Care Mental Health Services. Psychiatric services (Washington, D.C.), 72(3), 242–246. <https://doi.org/10.1176/appi.ps.202000467>
2. Aguilar, L., Vicente-Hernández, B., Remón-Gallo, D., García-Ullán, L., Valriberas-Herrero, I., Maciá-Casas, A., Pérez-Madruga, A., Garzón, M.A., Álvarez-Navares, A., Roncero, C. (2021). A real-world ten-week follow-up of the COVID outbreak in an outpatient drug clinic in Salamanca (Spain). J Subst Abuse Treat, 125: 108303.
3. Amad, A., Magnat, M., Quilès, C., Yrondi, A., Sauvaget, A., Bulteau, S., Plaze, M., Rotharmel, M., Polosan, M., Lévy-Chavagnat, D., Jaafari, N., Vaiva, G., & Thomas, P. (2020). Évolution de l’activité d’électro-convulsivo-thérapie en France depuis le début de la pandémie COVID-19 [Evolution of electro-convulsive therapy activity in France since the beginning of the COVID-19 pandemic]. L'Encephale, 46(3S), S40–S42. <https://doi.org/10.1016/j.encep.2020.04.004>.
4. Aragona, M., Barbato, A., Cavani, A., Costanzo, G., & Mirisola, C. (2020). Negative impacts of COVID-19 lockdown on mental health service access and follow-up adherence for immigrants and individuals in socio-economic difficulties. Public health, 186, 52–56. <https://doi.org/10.1016/j.puhe.2020.06.055>
5. Arendt, F., Markiewitz, A., Mestas, M., & Scherr, S. (2020). COVID-19 pandemic, government responses, and public mental health: Investigating consequences through crisis hotline calls in two countries. Social science & medicine (1982), 265, 113532. <https://doi.org/10.1016/j.socscimed.2020.113532>
6. Baugh, J. J., White, B. A., McEvoy, D., Yun, B. J., Brown, D., Raja, A. S., & Dutta, S. (2021). The cases not seen: Patterns of emergency department visits and procedures in the era of COVID-19. The American journal of emergency medicine, 46, 476–481. <https://doi.org/10.1016/j.ajem.2020.10.081>
7. Beran, C., & Sowa, N. A. (2021). Adaptation of an Academic Inpatient Consultation-Liaison Psychiatry Service During the SARS-CoV-2 Pandemic: Effects on Clinical Practice and Trainee Supervision. Journal of the Academy of Consultation-Liaison Psychiatry, 62(2), 186–192. <https://doi.org/10.1016/j.psym.2020.11.002>
8. Berthaut, E., & Chamignon, T. (2021). Le fil institutionnel en période Covid : utilisation de la plateforme Discord au CMPA. Neuropsychiatrie de l'enfance et de l'adolescence, 69, 3, 142-149. <https://doi.org/10.1016/j.neurenf.2021.01.002>.
9. Boldrini, T., Schiano Lomoriello, A., Del Corno, F., Lingiardi, V., & Salcuni, S. (2020). Psychotherapy During COVID-19: How the Clinical Practice of Italian Psychotherapists Changed During the Pandemic. Frontiers in psychology, 11, 591170. <https://doi.org/10.3389/fpsyg.2020.591170>.
10. Bollmann, A., Hohenstein, S., Pellissier, V., Stengler, K., Reichardt, P., Ritz, J. P., Thiele, H., Borger, M. A., Hindricks, G., Meier-Hellmann, A., & Kuhlen, R. (2021). Utilization of in- and outpatient hospital care in Germany during the Covid-19 pandemic insights from the German-wide Helios hospital network. PloS one, 16(3), e0249251. <https://doi.org/10.1371/journal.pone.0249251>
11. Butler, M., Delvi, A., Mujic, F., Broad, S., Pauli, L., Pollak, T. A., Gibbs, S., Fai Lam, C., Calcia, M. A., & Posporelis, S. (2021). Reduced Activity in an Inpatient Liaison Psychiatry Service During the First Wave of the COVID-19 Pandemic: Comparison With 2019 Data and Characterization of the SARS-CoV-2 Positive Cohort. Frontiers in psychiatry, 12, 619550. <https://doi.org/10.3389/fpsyt.2021.619550>
12. Cantini, S., Caponigro, G., Fusco, O., Deligiannis, P., Pastorini, A., Cubeddu, A., Maiolani, M., Menatti, E., Bazza, T., Bertolini, A. (2020). Psychoncology in front of covid-19. Tumori, 106, suppl 2, 95-96. <https://doi.org/10.1177/0300891620953388>.
13. Carpiniello, B., Tusconi, M., Zanalda, E. et al. (2020a). Psychiatry during the Covid-19 pandemic: a survey on mental health departments in Italy. BMC Psychiatry 20, 593. <https://doi.org/10.1186/s12888-020-02997-z>
14. Carpiniello, B., Tusconi, M., di Sciascio, G., Zanalda, E., di Giannantonio, M. (2020b) Mental health services in Italy during the COVID-19 pandemic. Psychiatry Clin Neurosci; 74(8): 442-443, 2020 08. <https://doi.org/10.1111/pcn.13082>.
15. Casano, K., & Capone, E. (2020). Unexpected cancellation on a catatonic patient's electroconvulsive therapy due to the coronavirus pandemic. General psychiatry, 33(6), e100271. <https://doi.org/10.1136/gpsych-2020-100271>.
16. Castelpietra, G., Colli, C., Tossut, D., Furlan, M., Balestrieri, M., Starace, F., Beghi, M., Barbone, F., Perulli, A., & Salvador-Carulla, L. (2021). The impact of Covid-19 pandemic on community-oriented mental health services: The experience of Friuli Venezia Giulia region, Italy. Health policy and technology, 10(1), 143–150. <https://doi.org/10.1016/j.hlpt.2020.12.002>
17. Chen, S., Jones, P. B., Underwood, B. R., Moore, A., Bullmore, E. T., Banerjee, S., Osimo, E. F., Deakin, J. B., Hatfield, C. F., Thompson, F. J., Artingstall, J. D., Slann, M. P., Lewis, J. R., & Cardinal, R. N. (2020). The early impact of COVID-19 on mental health and community physical health services and their patients' mortality in Cambridgeshire and Peterborough, UK. Journal of psychiatric research, 131, 244–254. <https://doi.org/10.1016/j.jpsychires.2020.09.020>
18. Clerici, M., Durbano, F., Spinogatti, F., Vita, A., de Girolamo, G., & Micciolo, R. (2020). Psychiatric hospitalization rates in Italy before and during COVID-19: did they change? An analysis of register data. Irish journal of psychological medicine, 37(4), 283–290. <https://doi.org/10.1017/ipm.2020.29>
19. Connolly, S. L., Stolzmann, K. L., Heyworth, L., Weaver, K. R., Bauer, M. S., & Miller, C. J. (2021). Rapid Increase in Telemental Health Within the Department of Veterans Affairs During the COVID-19 Pandemic. Telemedicine journal and e-health : the official journal of the American Telemedicine Association, 27(4), 454–458. <https://doi.org/10.1089/tmj.2020.0233>
20. Cui, Y., Li, Y., Zheng, Y., & Chinese Society of Child & Adolescent Psychiatry (2020). Mental health services for children in China during the COVID-19 pandemic: results of an expert-based national survey among child and adolescent psychiatric hospitals. European child & adolescent psychiatry, 29(6), 743–748. <https://doi.org/10.1007/s00787-020-01548-x>.
21. Datta, N., Derenne, J., Sanders, M., & Lock, J. D. (2020). Telehealth transition in a comprehensive care unit for eating disorders: Challenges and long-term benefits. The International journal of eating disorders, 53(11), 1774–1779. <https://doi.org/10.1002/eat.23348>
22. Di Lorenzo, R., Fiore, G., Bruno, A., Pinelli, M., Bertani, D., Falcone, P., Marrama, D., Starace, F., & Ferri, P. (2021). Urgent Psychiatric Consultations at Mental Health Center during COVID-19 Pandemic: Retrospective Observational Study. The Psychiatric quarterly, 92(4), 1341–1359. <https://doi.org/10.1007/s11126-021-09907-w>
23. Díaz de Neira, M., Blasco-Fontecilla, H., García Murillo, L., Pérez-Balaguer, A., Mallol, L., Forti, A., Del Sol, P., & Palanca, I. (2021). Demand Analysis of a Psychiatric Emergency Room and an Adolescent Acute Inpatient Unit in the Context of the COVID-19 Pandemic in Madrid, Spain. Frontiers in psychiatry, 11, 557508. <https://doi.org/10.3389/fpsyt.2020.557508>
24. Dolp, R. (2020). Assessing and managing children with urgent psychiatric needs during COVID-19. J Can Acad Child Adolesc Psychiatry, 29, 4.
25. Ferrando, S. J., Klepacz, L., Lynch, S., Shahar, S., Dornbush, R., Smiley, A., Miller, I., Tavakkoli, M., Regan, J., & Bartell, A. (2021). Psychiatric emergencies during the height of the COVID-19 pandemic in the suburban New York City area. Journal of psychiatric research, 136, 552–559. <https://doi.org/10.1016/j.jpsychires.2020.10.029>
26. Fredwall, M., Terry, D., Enciso, L., Burch, M. M., Trott, K., & Albert, D. (2021). Short-term outcomes in pediatric and adolescent patients with psychogenic nonepileptic events seen by telemedicine during the COVID-19 pandemic. Epilepsy & behavior : E&B, 117, 107739. <https://doi.org/10.1016/j.yebeh.2020.107739>
27. Garcia-Huidobro, D., Rivera, S., Valderrama Chang, S., Bravo, P., & Capurro, D. (2020). System-Wide Accelerated Implementation of Telemedicine in Response to COVID-19: Mixed Methods Evaluation. Journal of medical Internet research, 22(10), e22146. <https://doi.org/10.2196/22146>
28. Ghiretti, F., Gildoni, G., Grassi, G. M., Torricelli, L., Benassi, E., Bonaretti, E., Bonazzi, F., Borelli, S., Cagnolati, F., Covati, K., Errera, F., Finardi, V., Grisendi, R., Libanti, J., Lumia, R., Montanari, A., Morini, G., Pettinari, S., Peverini, A., Ragone, C., … Poletti, M. (2020). Psychological Support to the Community During the COVID-19 Pandemic: Field Experience in Reggio Emilia, Northern Italy. Frontiers in psychology, 11, 561742. <https://doi.org/10.3389/fpsyg.2020.561742>.
29. Ghosh, A., Mahintamani, T., B N, S., Pillai, R. R., Mattoo, S. K., & Basu, D. (2021). Telemedicine-assisted stepwise approach of service delivery for substance use disorders in India. Asian journal of psychiatry, 58, 102582. <https://doi.org/10.1016/j.ajp.2021.102582>
30. Giannouchos, T. V., Biskupiak, J., Moss, M. J., Brixner, D., Andreyeva, E., & Ukert, B. (2021). Trends in outpatient emergency department visits during the COVID-19 pandemic at a large, urban, academic hospital system. The American journal of emergency medicine, 40, 20–26. <https://doi.org/10.1016/j.ajem.2020.12.009>
31. Gómez-Ramiro, M., Fico, G., Anmella, G., Vázquez, M., Sagué-Vilavella, M., Hidalgo-Mazzei, D., Pacchiarotti, I., Garriga, M., Murru, A., Parellada, E., & Vieta, E. (2021). Changing trends in psychiatric emergency service admissions during the COVID-19 outbreak: Report from a worldwide epicentre. Journal of affective disorders, 282, 26–32. <https://doi.org/10.1016/j.jad.2020.12.057>
32. Graell, M., Morón-Nozaleda, M. G., Camarneiro, R., Villaseñor, Á., Yáñez, S., Muñoz, R., Martínez-Núñez, B., Miguélez-Fernández, C., Muñoz, M., & Faya, M. (2020). Children and adolescents with eating disorders during COVID-19 confinement: Difficulties and future challenges. European eating disorders review : the journal of the Eating Disorders Association, 28(6), 864–870. <https://doi.org/10.1002/erv.2763>
33. Grover, S., Mehra, A., Sahoo, S., Avasthi, A., Tripathi, A., D'Souza, A., Saha, G., Jagadhisha, A., Gowda, M., Vaishnav, M., Singh, O., Dalal, P. K., & Kumar, P. (2020b). State of mental health services in various training centers in India during the lockdown and COVID-19 pandemic. Indian journal of psychiatry, 62(4), 363–369. <https://doi.org/10.4103/psychiatry.IndianJPsychiatry_567_20>.
34. Grover, S., Mehra, A., Sahoo, S., Avasthi, A., Tripathi, A., D'Souza, A., Saha, G., Jagadhisha, A., Gowda, M., Vaishnav, M., Singh, O., Dalal, P. K., & Kumar, P. (2020c). Impact of COVID-19 pandemic and lockdown on the state of mental health services in the private sector in India. Indian journal of psychiatry, 62(5), 488–493. <https://doi.org/10.4103/psychiatry.IndianJPsychiatry_568_20>.
35. Grunvald, W., Herrington, R., King, R., Lamberson, M., Mackey, S., Maruti, S., Rawson, R., & Wolfson, D. (2021). COVID-19: A new barrier to treatment for opioid use disorder in the emergency department. Journal of the American College of Emergency Physicians open, 2(2), e12403. <https://doi.org/10.1002/emp2.12403>
36. Guinart, D., Marcy, P., Hauser, M., Dwyer, M., & Kane, J. M. (2021). Mental Health Care Providers' Attitudes Toward Telepsychiatry: A Systemwide, Multisite Survey During the COVID-19 Pandemic. Psychiatric services (Washington, D.C.), 72(6), 704–707. <https://doi.org/10.1176/appi.ps.202000441>.
37. Håkansson, A., Åkesson, G., Grudet, C., & Broman, N. (2021). No Apparent Increase in Treatment Uptake for Gambling Disorder during Ten Months of the COVID-19 Pandemic-Analysis of a Regional Specialized Treatment Unit in Sweden. International journal of environmental research and public health, 18(4), 1918. <https://doi.org/10.3390/ijerph18041918>
38. Hall, P., & Sukhera, J. (2020). Emergency Diversion Clinic: Lessons from Southwestern Ontario's Pandemic Response. Journal of the Canadian Academy of Child and Adolescent Psychiatry, 29, 4, 271.
39. Hames, J. L., Bell, D. J., Perez-Lima, L. M., Holm-Denoma, J. M., Rooney, T., Charles, N. E., Thompson, S. M., Mehlenbeck, R. S., Tawfik, S. H., Fondacaro, K. M., Simmons, K. T., Hoersting, R. C. (2020). Navigating uncharted waters: Considerations for training clinics in the rapid transition to telepsychology and telesupervision during COVID-19. Journal of Psychotherapy Integration, 30, 2, 348-365. <https://doi.org/10.1037/int0000224>
40. Harrison, L., DeMasi, L., Butkus, S., O'Neill, K., Kwon, N., D'Angelo, J., Morgenstern, J., & Kapoor, S. (2020). 163 Implementation of a Telephonic-Based Model to Continue to Address Substance Use as Part of Usual Care in Emergency Departments during COVID-19. Annals of Emergency Medicine, 76(4), S64. <https://doi.org/10.1016/j.annemergmed.2020.09.175>.
41. Hazarika, M., Das, B., Das, S., Baruah, A., Sharma, N., Barua, C., Das, J., Choudhury, S., Hazarika, D., Sarma, P., & Bhandari, S. S. (2021). Profile of distress callers and service utilisation of tele-counselling among the population of Assam, India: an exploratory study during COVID-19. Open journal of psychiatry & allied sciences, 12(1), 7–12. <https://doi.org/10.5958/2394-2061.2021.00001.x>.
42. Herring, A., Kalmin, M., Speener, M., Goodman-Meza, D., Snyder, H., Campbell, A., et al. (2021). Sharp decline in hospital and emergency department initiated buprenorphine for opioid use disorder during COVID-19 state of emergency in California. UCLA. Report #: ARTN 108260. http://dx.doi.org/10.1016/j.jsat.2020.108260 Retrieved from <https://escholarship.org/uc/item/6xq102k9>
43. Hoffnung, G., Feigenbaum, E., Schechter, A., Guttman, D., Zemon, V., Schechter, I. (2020). Children and Telehealth in Mental Healthcare: What We Have Learned From COVID‐19 and 40,000+ Sessions. Psych res clin pract, <https://doi.org/10.1176/appi.prcp.20200035>
44. Hoyer, C., Ebert, A., Szabo, K., Platten, M., Meyer-Lindenberg, A., & Kranaster, L. (2021). Decreased utilization of mental health emergency service during the COVID-19 pandemic. European archives of psychiatry and clinical neuroscience, 271(2), 377–379. <https://doi.org/10.1007/s00406-020-01151-w>
45. Humer, E., Pieh, C., Kuska, M., Barke, A., Doering, B. K., Gossmann, K., Trnka, R., Meier, Z., Kascakova, N., Tavel, P., & Probst, T. (2020a). Provision of Psychotherapy during the COVID-19 Pandemic among Czech, German and Slovak Psychotherapists. International journal of environmental research and public health, 17(13), 4811. <https://doi.org/10.3390/ijerph17134811>.
46. Itrat, A., Jagadheesan, K., Danivas, V., & Lakra, V. (2020). A comparative study of access to inpatient psychiatric treatment in a public mental health service in Melbourne during COVID-19. Indian journal of psychiatry, 62(Suppl 3), S454–S458. <https://doi.org/10.4103/psychiatry.IndianJPsychiatry_852_20>
47. Jagadheesan, K., Danivas, V., Itrat, Q., Sekharan, L., & Lakra, A. (2021). COVID-19 and psychiatric admissions: An observational study of the first six months of lockdown in Melbourne. Psychiatry research, 300, 113902. <https://doi.org/10.1016/j.psychres.2021.113902>
48. Johnson, S., Dalton-Locke, C., Vera San Juan, N., Foye, U., Oram, S., Papamichail, A., Landau, S., Rowan Olive, R., Jeynes, T., Shah, P., Sheridan Rains, L., Lloyd-Evans, B., Carr, S., Killaspy, H., Gillard, S., Simpson, A., & COVID-19 Mental Health Policy Research Unit Group (2020a). Impact on mental health care and on mental health service users of the COVID-19 pandemic: a mixed methods survey of UK mental health care staff. Social psychiatry and psychiatric epidemiology, 56(1), 25–37. <https://doi.org/10.1007/s00127-020-01927-4>.
49. Johnson, E., Bluthenthal, R., Trotsky-Sirr, R., Schneberk, T. (2020b) 57EMF X-Waiver Training for Resident Physicians Increases Emergency Department Buprenorphine Delivery: An Implementation Science Evaluation. Annals of Emergency Medicine, Research forum abstract, 76, 4, s23. <https://doi.org/10.1016/j.annemergmed.2020.09.067>
50. Karim, M. A., Wadoo, O., Reagu, S. M., Amro, R., & Abdulla, M. A. (2020). Telepsychiatry in the Arabian Gulf region - Implications beyond the Covid-19 pandemic. Asian journal of psychiatry, 54, 102397. https://doi.org/10.1016/j.ajp.2020.102397
51. Khanra, S., Mukherjee, A., Goyal, N., Das, B., & Munda, S. K. (2021). Service utilization and saved travel cost in telepsychiatry consultation by outpatients at a psychiatric hospital in India during COVID-19 pandemic. Asian journal of psychiatry, 57, 102568. <https://doi.org/10.1016/j.ajp.2021.102568>.
52. Kuitunen, I., Ponkilainen, V. T., Launonen, A. P., Reito, A., Hevonkorpi, T. P., Paloneva, J., & Mattila, V. M. (2020). The effect of national lockdown due to COVID-19 on emergency department visits. Scandinavian journal of trauma, resuscitation and emergency medicine, 28(1), 114. <https://doi.org/10.1186/s13049-020-00810-0>
53. Lee, A.T.C., Mo, F.Y.M., Lam, L.C.W. (2020). Higher psychogeriatric admissions in COVID-19 than in severe acute respiratory syndrome. International journal of Geriatric psychiatry, 35, 12, 1449-1457. <https://doi.org/10.1002/gps.5422>
54. Li, L., Roberts, S. C., Kulp, W., Wing, A., Barnes, T., Colandrea, N., Klink, B., Fortunati, F., & Martinello, R. (2021). Epidemiology, Infection Prevention, Testing Data, and Clinical Outcomes of COVID-19 on Five Inpatient Psychiatric Units in a large Academic Medical Center. Psychiatry research, 298, 113776. <https://doi.org/10.1016/j.psychres.2021.113776>
55. Lian, W., Wen, L., Zhou, Q., Zhu, W., Duan, W., Xiao, X., Mhungu, F., Huang, W., Li, C., Cheng, W., & Tian, J. (2020). Digital Health Technologies Respond to the COVID-19 Pandemic In a Tertiary Hospital in China: Development and Usability Study. Journal of medical Internet research, 22(11), e24505. <https://doi.org/10.2196/24505>
56. Looi, J. C., Allison, S., Bastiampillai, T., Pring, W., & Reay, R. (2020a). Australian private practice metropolitan telepsychiatry during the COVID-19 pandemic: analysis of Quarter-2, 2020 usage of new MBS-telehealth item psychiatrist services. Australasian Psychiatry, 29(2), 183–188. <https://doi.org/10.1177/1039856220975294>
57. Looi, J. C., Allison, S., Bastiampillai, T., & Pring, W. (2020b). Private practice metropolitan telepsychiatry in smaller Australian jurisdictions during the COVID-19 pandemic: preliminary analysis of the introduction of new Medicare Benefits Schedule items. Australasian psychiatry : bulletin of Royal Australian and New Zealand College of Psychiatrists, 28(6), 639–643. <https://doi.org/10.1177/1039856220960381>
58. Looi, J. C., Allison, S., Bastiampillai, T., & Pring, W. (2020d). Private practice metropolitan telepsychiatry in larger Australian states during the COVID-19 pandemic: an analysis of the first 2 months of new MBS telehealth item psychiatrist services. Australasian Psychiatry, 28(6), 644–648. <https://doi.org/10.1177/1039856220961906>
59. Looi, J. C., Allison, S., Bastiampillai, T., Pring, W., Reay, R., & Kisely, S. R. (2021). Increased Australian outpatient private practice psychiatric care during the COVID-19 pandemic: usage of new MBS-telehealth item and face-to-face psychiatrist office-based services in Quarter 3, 2020. Australasian psychiatry : bulletin of Royal Australian and New Zealand College of Psychiatrists, 29(2), 194–199. <https://doi.org/10.1177/1039856221992634>
60. Lunsky, Y., Bobbette, N., Selick, A., & Jiwa, M. I. (2021). "The doctor will see you now": Direct support professionals' perspectives on supporting adults with intellectual and developmental disabilities accessing health care during COVID-19. Disability and health journal, 14(3), 101066. <https://doi.org/10.1016/j.dhjo.2021.101066>.
61. Makiyama, K., Kawashima, T., Nomura, S., Eguchi, A., Yoneoka, D., Tanoue, Y., Kawamura, Y., Sakamoto, H., Gilmour, S., Shi, S., Matsuura, K., Uryu, S., & Hashizume, M. (2021). Trends in Healthcare Access in Japan during the First Wave of the COVID-19 Pandemic, up to June 2020. International journal of environmental research and public health, 18(6), 3271. <https://doi.org/10.3390/ijerph18063271>
62. Martin, C., Andrés, P., Bullón, A., Villegas, J. L., de la Iglesia-Larrad, J. I., Bote, B., Prieto, N., & Roncero, C. (2021). COVID pandemic as an opportunity for improving mental health treatments of the homeless people. The International journal of social psychiatry, 67(4), 335–343. <https://doi.org/10.1177/0020764020950770>.
63. Mehtani, N. J., Ristau, J. T., Snyder, H., Surlyn, C., Eveland, J., Smith-Bernardin, S., & Knight, K. R. (2021). COVID-19: A catalyst for change in telehealth service delivery for opioid use disorder management. Substance abuse, 42(2), 205–212. <https://doi.org/10.1080/08897077.2021.1890676>
64. Mishkind, M. C., Shore, J. H., Bishop, K., D'Amato, K., Brame, A., Thomas, M., & Schneck, C. D. (2021). Rapid Conversion to Telemental Health Services in Response to COVID-19: Experiences of Two Outpatient Mental Health Clinics. Telemedicine journal and e-health: the official journal of the American Telemedicine Association, 27(7), 778–784. <https://doi.org/10.1089/tmj.2020.0304>
65. Moreland, A., Guille, C., & McCauley, J. L. (2021). Increased availability of telehealth mental health and substance abuse treatment for peripartum and postpartum women: A unique opportunity to increase telehealth treatment. Journal of substance abuse treatment, 123, 108268. <https://doi.org/10.1016/j.jsat.2020.108268>
66. Mustafa, F. A. (2021). No reduction in psychiatric emergency department visits during COVID-19: A downside of telepsychiatry? Journal of Marketing, 67(4), 17–32. <https://doi.org/10.1509/jm.11.0423>
67. Myers Virtue, S., Howrey, H. L., Duffy, N. M., & Wallace, M. (2021). Shifting psychosocial oncology care to telepsychology during the COVID-19 pandemic. Journal of psychosocial oncology, 39(3), 416–427. <https://doi.org/10.1080/07347332.2021.1894526>.
68. Naik, S. S., Rahul, P., Harihara, S., Pahuja, E., Chithra, N. K., Ramachandraiah, S., Kumar, V., Mehta, U. M., Manjunatha, N., Channaveerachari, N. K., Math, S. B., & Thirthalli, J. (2021). Telephonic follow-up during COVID-19 to maintain continuity of care for persons with psychiatric disorders. Asian journal of psychiatry, 57, 102564. <https://doi.org/10.1016/j.ajp.2021.102564>.
69. Peppou, L. E., Economou, M., Skali, T., & Papageorgiou, C. (2021). From economic crisis to the COVID-19 pandemic crisis: evidence from a mental health helpline in Greece. European archives of psychiatry and clinical neuroscience, 271(2), 407–409. <https://doi.org/10.1007/s00406-020-01165-4>
70. Peralta, E. A., & Taveras, M. (2020). Effectiveness of teleconsultation use in access to mental health services during the coronavirus disease 2019 pandemic in the Dominican Republic. Indian journal of psychiatry, 62(Suppl 3), S492–S494. <https://doi.org/10.4103/psychiatry.IndianJPsychiatry_1047_20>.
71. Perricone, G., Rotolo, I., Beninati, V., Billeci, N., Ilarda, V., & Polizzi, C. (2021). The Lègami/Legàmi Service-An Experience of Psychological Intervention in Maternal and Child Care during COVID-19. Pediatric reports, 13(1), 142–161. <https://doi.org/10.3390/pediatric13010021>.
72. Pham-Scottez, A., Silva, J., Barruel, D., Masson, V. D., Yon, L., Trebalag, A. K., & Gourevitch, R. (2020). Patient flow in the largest French psychiatric emergency centre in the context of the COVID-19 pandemic. Psychiatry research, 291, 113205. <https://doi.org/10.1016/j.psychres.2020.113205>
73. Pierce, B. S., Perrin, P. B., Tyler, C. M., McKee, G. B., & Watson, J. D. (2021). The COVID-19 telepsychology revolution: A national study of pandemic-based changes in U.S. mental health care delivery. The American psychologist, 76(1), 14–25. <https://doi.org/10.1037/amp0000722>.
74. Prior, M., Bellè, M., Burelli, M., Favaretto, A. (2020). On-line psychological treatment with oncology patients during COVID-19 emergency. Tumori, 106, suppl 2, 81. <https://doi.org/10.1177/0300891620953388>.
75. Probst, T., Stippl, P., & Pieh, C. (2020). Changes in Provision of Psychotherapy in the Early Weeks of the COVID-19 Lockdown in Austria. International journal of environmental research and public health, 17(11), 3815. <https://doi.org/10.3390/ijerph17113815>.
76. Rainwater, L., Lichiello, S., Duckworth, K., Tolbert, A., Moskop, R., McQuellon, R. (2021). Psychosocial oncology care during a pandemic: Effect of offering telemental health in an academic medical center. Psycho-oncology, 30, S1, 18.1. <http://doi:10.1002/pon.5637>
77. Ravindran, S., P, L. N., Channaveerachari, N. K., Seshadri, S. P., Kasi, S., Manikappa, S. K., Cherian, A. V., Palanimuthu T, S., Sudhir, P., Govindan, R., P, B. R., Christopher, A. D., & George, S. (2020). Crossing barriers: Role of a tele-outreach program addressing psychosocial needs in the midst of COVID-19 pandemic. Asian journal of psychiatry, 53, 102351. <https://doi.org/10.1016/j.ajp.2020.102351>.
78. Roncero, C., García-Ullán, L., de la Iglesia-Larrad, J. I., Martín, C., Andrés, P., Ojeda, A., González-Parra, D., Pérez, J., Fombellida, C., Álvarez-Navares, A., Benito, J. A., Dutil, V., Lorenzo, C., & Montejo, Á. L. (2020a). The response of the mental health network of the Salamanca area to the COVID-19 pandemic: The role of the telemedicine. Psychiatry research, 291, 113252. <https://doi.org/10.1016/j.psychres.2020.113252>
79. Roncero, C., Vicente-Hernández, B., Casado-Espada, N. M., Aguilar, L., Gamonal-Limcaoco, S., Garzón, M. A., Martínez-González, F., Llanes-Álvarez, C., Martínez, R., Franco-Martín, M., & Álvarez-Navares, A. (2020). The Impact of COVID-19 Pandemic on the Castile and Leon Addiction Treatment Network: A Real-Word Experience. Frontiers in psychiatry, 11, 575755. <https://doi.org/10.3389/fpsyt.2020.575755>.
80. Rosen, C. S., Morland, L. A., Glassman, L. H., Marx, B. P., Weaver, K., Smith, C. A., Pollack, S., & Schnurr, P. P. (2021). Virtual mental health care in the Veterans Health Administration's immediate response to coronavirus disease-19. The American psychologist, 76(1), 26–38. <https://doi.org/10.1037/amp0000751>
81. Saberian, P., Conovaloff, J. L., Vahidi, E., Hasani-Sharamin, P., & Kolivand, P. H. (2020). How the COVID-19 Epidemic Affected Prehospital Emergency Medical Services in Tehran, Iran. The western journal of emergency medicine, 21(6), 110–116. <https://doi.org/10.5811/westjem.2020.8.48679>
82. Salum, G. A., Rehmenklau, J. F., Csordas, M. C., Pereira, F. P., Castan, J. U., Ferreira, A. B., Delgado, V. B., Bolzan, L. M., de Lima, M. A., Blauth, J. H., Dos Reis, J. R., Rocha, P. B., Guerra, T. A., Saraiva, I. M., Gramz, B. C., Ronchi, B. R., Ribeiro, B. L., Konig, D. F., & Grevet, E. H. (2020). Supporting people with severe mental health conditions during the COVID-19 pandemic: considerations for low- and middle-income countries using telehealth case management. Revista brasileira de psiquiatria (Sao Paulo, Brazil : 1999), 42(4), 451–452. <https://doi.org/10.1590/1516-4446-2020-1078>
83. Sampaio, M., Haro, M., De Sousa, B., Melo, W. V., & Hoffman, H. G. (2021). Therapists Make the Switch to Telepsychology to Safely Continue Treating Their Patients During the COVID-19 Pandemic. Virtual Reality Telepsychology May Be Next. Frontiers in virtual reality, 1, 576421. <https://doi.org/10.3389/frvir.2020.576421>.
84. Schreiber, S., Tene, O., Mordel, C., Sason, A., & Peles, E. (2021). A decrease in psychiatric consultations at the emergency room and inpatient wards of a large general hospital in Israel during the SARS-CoV-2 (COVID-19) pandemic. General hospital psychiatry, 70, 145–146. <https://doi.org/10.1016/j.genhosppsych.2021.02.005>
85. Sharma, A., Sasser, T., Schoenfelder Gonzalez, E., Vander Stoep, A., & Myers, K. (2020). Implementation of Home-Based Telemental Health in a Large Child Psychiatry Department During the COVID-19 Crisis. Journal of child and adolescent psychopharmacology, 30(7), 404–413. <https://doi.org/10.1089/cap.2020.0062>
86. Singh Bhandari, S., Joseph, S. J., Udayasankaran, J. G., Konthoujam, B., Shoib, S., & Dutta, S. (2020). Telepsychiatry: A Feasible Means to Bridge the Demand-Supply Gaps in Mental Health Services During and After the COVID-19 Pandemic: Preliminary Experiences from Sikkim State of India. Indian journal of psychological medicine, 42(5), 500–502. <https://doi.org/10.1177/0253717620951282>.
87. Spalletta, G., Porcari, D. E., Banaj, N., Ciullo, V., & Palmer, K. (2020). Effects of COVID-19 Infection Control Measures on Appointment Cancelation in an Italian Outpatient Memory Clinic. Frontiers in psychiatry, 11, 599844. <https://doi.org/10.3389/fpsyt.2020.599844>
88. Staples, L., Nielssen, O., Kayrouz, R., Cross, S., Karin, E., Ryan, K., Dear, B., & Titov, N. (2020). Rapid report 2: Symptoms of anxiety and depression during the first 12 weeks of the Coronavirus (COVID-19) pandemic in Australia. Internet interventions, 22, 100351. <https://doi.org/10.1016/j.invent.2020.100351>
89. Steeg, S., Carr, M.J., Trefan, L., Ashcroft, D/M., Kapur, N., Nielsen, E., McMillan, B., Webb, R.T. (2021). Primary care clinical management following self-harm during the first wave of COVID-19 in the UK. medRxiv 2021.03.19.21253969. <https://doi.org/10.1101/2021.03.19.21253969>
90. Stewart, R. & Broadbent, M. (2020). Using past and current data to estimate potential crisis service use in mental healthcare after the COVID-19 lockdown: South London and Maudsley data medRxiv 2020.06.29.20142448. <https://doi.org/10.1101/2020.06.29.20142448>.
91. Stewart, R., Martin, E., Broadbent, M. (2020). Mental health service activity during COVID-19 lockdown: South London and Maudsley data on working age community and home treatment team services and mortality from February to mid-May 2020. medRxiv 2020.06.13.20130419. <https://doi.org/10.1101/2020.06.13.20130419>
92. Sutherland, K., Chessman, J., Zhao, J., Sara, G., Shetty, A., Smith, S., Went, A., Dyson, S., & Levesque, J. F. (2020). Impact of COVID-19 on healthcare activity in NSW, Australia. Public health research & practice, 30(4), 3042030. <https://doi.org/10.17061/phrp3042030>
93. Sveticic, J., Stapelberg, N. J., & Turner, K. (2021). Suicide prevention during COVID-19: identification of groups with reduced presentations to emergency departments. Australasian psychiatry: bulletin of Royal Australian and New Zealand College of Psychiatrists, 29(3), 333–336. <https://doi.org/10.1177/1039856221992632>
94. Tringale, R., & Subica, A. M. (2021). COVID-19 innovations in medication for addiction treatment at a Skid Row syringe exchange. Journal of substance abuse treatment, 121, 108181. <https://doi.org/10.1016/j.jsat.2020.108181>
95. Turan, Ş., Poyraz, B. Ç., Aksoy Poyraz, C., Demirel, Ö. F., Tanrıöver Aydın, E., Uçar Bostan, B., Demirel, Ö., & Ali, R. K. (2021). Characteristics and outcomes of COVID-19 inpatients who underwent psychiatric consultations. Asian journal of psychiatry, 57, 102563. <https://doi.org/10.1016/j.ajp.2021.102563>.
96. Ugueto, A. M., & Zeni, C. P. (2021). Patterns of Youth Inpatient Psychiatric Admissions Before and After the Onset of the COVID-19 Pandemic. Journal of the American Academy of Child and Adolescent Psychiatry, 60(7), 796–798. <https://doi.org/10.1016/j.jaac.2021.02.006>
97. Vonderlin, R., Biermann, M., Konrad, M., Klett, M., Kleindienst, N., Bailer, J., Lis, S., & Bohus, M. (2021). Implementierung und Evaluation einer Telefonhotline zur professionellen Ersthilfe bei psychischen Belastungen durch die COVID-19-Pandemie in Baden-Württemberg [Implementation and evaluation of a telephone hotline for professional mental health first aid during the COVID-19 pandemic in Germany]. Der Nervenarzt, 93(1), 24–33. Epub Mar 2021. <https://doi.org/10.1007/s00115-021-01089-x>
98. Yaffa, S., Adi, E. L., Itai, P., Marit, J. M., Doron, G., & Daniel, S. (2021). Treatment of eating disorders in adolescents during the COVID-19 pandemic: a case series. Journal of eating disorders, 9(1), 17. <https://doi.org/10.1186/s40337-021-00374-z>
99. Yang, J., Landrum, M. B., Zhou, L., & Busch, A. B. (2020). Disparities in outpatient visits for mental health and/or substance use disorders during the COVID surge and partial reopening in Massachusetts. General hospital psychiatry, 67, 100–106. <https://doi.org/10.1016/j.genhosppsych.2020.09.004>
100. Zhong, B. L., Zhou, D. Y., He, M. F., Li, Y., Li, W. T., Ng, C. H., Xiang, Y. T., & Chiu, H. F. (2020). Mental health problems, needs, and service use among people living within and outside Wuhan during the COVID-19 epidemic in China. Annals of translational medicine, 8(21), 1392. <https://doi.org/10.21037/atm-20-4145>.
101. Zulfic, Z., Liu, D., Lloyd, C., Rowan, J., & Schubert, K. O. (2020). Is telepsychiatry care a realistic option for community mental health services during the COVID-19 pandemic? The Australian and New Zealand journal of psychiatry, 54(12), 1228. <https://doi.org/10.1177/0004867420937788>.
