# Supplementary material for: Impact of the COVID-19 Pandemic on the Global Delivery of Mental Health Services and Telemental Health: Systematic Review
Source: JMIR Ment Health. 2022 Aug 22;9(8):e38600. doi: 10.2196/38600 (PMC9400843; doi:10.2196/38600)
Supplement: Multimedia Appendix 5 [file mental_v9i8e38600_app5.docx]

**Multimedia Appendix 5.** Characteristics of 72 papers on face-to-face services.

| Study ID | Study design | Country | Data, start date | Data, end date |
| --- | --- | --- | --- | --- |
| Abbas et al. 2020 | Before-after study | United Kingdom | 16-3-20 | 16-4-20 |
| Aguilar et al. 2021 | Before-after study | Spain | 16-3-20 | 22-5-20 |
| Amad et al. 2020 | Survey | France | unknown | unknown |
| Aragona et al. 2020 | Before-after study | Italy | 1-2-20 | 31-3-20 |
| Baugh et al. 2020 | Before-after study | USA | 1-3-20 | 30-4-20 |
| Beran et al. 2020 | Before-after study | USA | 2-1-20 | 6-6-20 |
| Boldrini et al. 2020 | Survey | Italy | 5-4-20 | 10-5-20 |
| Bollmann et al. 2021 | Before-after study | Germany | 13-3-20 | 10-12-20 |
| Butler et al. 2021 | Before-after study | United Kingdom | 2-3-20 | 10-5-20 |
| Carpiniello et al. 2020a | Survey | Italy | 1-4-20 | 11-4-20 |
| Carpiniello et al. 2020b (secondary of Carpiniello 2020a) | Survey | Italy | 1-4-20 | 11-4-20 |
| Casano et al. 2020 | Case report/case series | USA | unknown | unknown |
| Castelpietra et al. 2021 | Before-after study | Italy | 1-1-20 | 30-4-20 |
| Chen et al. 2020b | Before-after study | United Kingdom | unknown | unknown |
| Clerici et al. 2020 | Before-after study | Italy | 21-2-20 | 31-3-20 |
| Cui et al. 2020 | Survey | China | 20-2-20 | 3-3-20 |
| Di Lorenzo et al. 2021 | Before-after study | Italy | 1-3-20 | 31-8-20 |
| Diaz de Neira et al. 2021 | Before-after study | Spain | 11-3-20 | 11-4-20 |
| Dolp et al. 2020 | Before-after study | Canada | 1-3-20 | 31-5-20 |
| Ferrando et al. 2020 | Before-after study | USA | 1-3-20 | 30-4-20 |
| Fredwall et al. 2021 | Before-after study | USA | 1-1-20 | 30-6-20 |
| Ghosh et al. 2021 | Before-after study | India | 18-5-20 | 31-8-20 |
| Giannouchos et al. 2020 | Before-after study | USA | 1-4-20 | 30-4-20 |
| Gomez-Ramiro et al. 2021 | Before-after study | Spain | 14-12-19 | 12-6-20 |
| Graell et al. 2020 | Case report/case series | Spain | 16-3-20 | 10-5-20 |
| Grover et al. 2020b | Survey | India | 1-5-20 | 18-5-20 |
| Grover et al. 2020c | Survey | India | 1-5-20 | 15-5-20 |
| Grunvald et al. 2021 | Before-after study | USA | 1-3-20 | 31-5-20 |
| Hakansson et al. 2021 | Before-after study | Sweden | 1-3-20 | 31-12-20 |
| Hall & Sukhera 2020 | Case report/case series | Canada | unknown | unknown |
| Hames et al. 2020 | Survey | USA | 11-3-20 | 31-3-20 |
| Herring et al. 2021 | Before-after study | USA | 1-5-19 | 30-4-20 |
| Hoffnung et al. 2021 | Before-after study | USA | 1-1-20 | 30-6-20 |
| Hoyer et al. 2020 | Before-after study | Germany | 1-1-20 | 19-4-20 |
| Humer et al. 2020a | Survey | Germany, Czech Republic, Slovakia | 24-3-20 | 20-5-20 |
| Itrat et al. 2021 | Before-after study | Australia | 16-3-20 | 12-5-20 |
| Jagadheesan et al. 2021 | Before-after study | Australia | 16-3-20 | 16-9-20 |
| Johnson et al. 2020a | Survey | USA | 1-1-20 | 1-5-20 |
| Johnson et al. 2020b | Before-after study | Taiwan | 1-3-20 | unknown |
| Kuitunen et al. 2020 | Before-after study | Finland | 1-2-20 | 30-4-20 |
| Lee et al. 2020 | Before-after study | Hong Kong | 1-11-19 | 30-4-20 |
| Li et al. 2021b | Before-after study | USA | 1-3-20 | 8-7-20 |
| Looi et al. 2020a | Before-after study | Australia | 1-4-20 | 30-6-20 |
| Looi et al. 2020b | Before-after study | Australia | 1-4-20 | 31-5-20 |
| Looi et al. 2020d | Before-after study | Australia | 1-4-20 | 31-5-20 |
| Looi et al. 2021 | Before-after study | Australia | 1-7-20 | 30-9-20 |
| Makiyama et al. 2021 | Before-after study | Japan | 1-1-20 | 30-6-20 |
| Martin et al. 2020 | Case report/case series | Spain | 27-3-20 | 25-5-20 |
| Mustafa et al. 2020 | Before-after study | United Kingdom | 1-3-20 | 30-4-20 |
| Pham-Scottez et al. 2020 | Before-after study | France | 17-3-20 | 10-5-20 |
| Probst et al. 2020 | Survey | Austria | 24-3-20 | 1-4-20 |
| Rainwater et al. 2020 | Before-after study | USA | 1-4-20 | 30-9-20 |
| Roncero et al. 2020a | Before-after study | Spain | unknown | unknown |
| Roncero et al. 2020b | Survey | Spain | unknown | unknown |
| Rosen et al. 2020 | Before-after study | USA | 1-10-19 | 30-6-20 |
| Saberian et al. 2020 | Before-after study | Iran | 21-1-20 | 18-3-20 |
| Salum et al. 2020 | Case report/case series | Brazil | 23-3-20 | 23-3-20 |
| Schreiber et al. 2021 | Before-after study | Israel | 1-1-20 | 21-12-20 |
| Sharma et al. 2020 | Case report/case series | USA | 28-2-20 | 3-4-20 |
| Spalletta et al. 2020 | Before-after study | Italy | 1-1-20 | 30-4-20 |
| Steeg et al. 2021 | Before-after study | United Kingdom | 10-3-20 | 10-6-20 |
| Stewart and Broadbent 2020 | Before-after study | United Kingdom | 16-2-20 | 16-4-20 |
| Stewart et al. 2020 | Before-after study | United Kingdom | 1-2-20 | 15-5-20 |
| Sutherland et al. 2020 | Before-after study | Australia | 1-3-20 | 30-6-20 |
| Sveticic et al. 2021 | Before-after study | Australia | 1-3-20 | 31-8-20 |
| Tringale et al. 2020 | Case report/case series | USA | 7-1-20 | 26-5-20 |
| Turan et al. 2021 | Case report/case series | Turkey | 10-3-20 | 26-6-20 |
| Ugueto et al. 2021 | Before-after study | USA | 11-3-20 | 30-8-20 |
| Yaffa et al. 2021 | Before-after study | Israel | 1-1-20 | 31-10-20 |
| Yang et al. 2020 | Before-after study | USA | 25-3-20 | 18-5-20 |
| Zhong et al. 2020 | Survey | China | 27-1-20 | 2-2-20 |
| Zulfic et al. 2020 | Survey | Australia | unknown | unknown |
